# An Empirical Study on the Influence of the Basic Medical Insurance for Urban and Rural Residents on Family Financial Asset Allocation

**DOI:** 10.3389/fpubh.2021.725608

**Published:** 2021-08-12

**Authors:** Shenglin Li, Yifei Yang

**Affiliations:** School of Insurance, Central University of Finance and Economics, Beijing, China

**Keywords:** basic medical insurance, urban and rural residents, family financial asset, asset allocation, heckman two-step method

## Abstract

This paper empirically examined whether participation in the Basic Medical Insurance for Urban and Rural Residents impacted families' allocation to risk assets and risk-free assets using the Heckman two-step method, which is based on the China Household Finance Survey micro data of 2013, 2015, and 2017. The results showed that participation in the Basic Medical Insurance for Urban and Rural Residents can promote families' reasonable choice between risk assets and risk-free assets to a certain extent. To be specific, the risk asset investments are squeezed out for the originally risk-seeking families, while the risk-free asset investments are squeezed out for the originally risk-adverse families. We tested the robustness of the benchmark model and the mediating effect model with different definitions of risk assets and risk-free assets. Also, the analysis of the mechanism showed that this increases families' risk perception—turning their risk attitude more cautious and their investment attitude more rational. To further consolidate the social security attributes of the Basic Medical Insurance for Urban and Rural Residents, behind its high coverage, we should also pay attention to its influence on the investment preferences of families with different social and economic statuses, thereby giving full play to its role in promoting the development of China's financial market. In future research, we can also try to use measurement models such as PSM-DID models, and find the connections and progressive relations between different models, in order to obtain the inquiry results of different dimensions. For the direction of further research in the future, we believe that can be used to test whether the conclusion whose data configuration of the basic medical insurance for family financial assets choice influence is a universal in developing countries, to explore the developing countries to promote the health security system for the influence of its national household financial asset allocation and the corresponding policy recommendations.

**JEL Classification:** D14, G11, H55, I18.

## Introduction

With the gradual promotion of a market-oriented reform, China's financial market continues to release new vitality and financial products, and allocable combinations are further enriched and diversified. The asset choice and allocation behavior of a family, which is an important basic unit of economy and society, plays a major role in the long-term living standard of the family; thus, the importance of the effective allocation of family financial assets is becoming increasingly prominent. According to the classical investment theory, a family will choose to allocate some of its assets to risky financial assets to realize the best balance between risk and return. However, contrary to the traditional theory, some families do not participate in perilous financial market investments, even if the participating investment does not hold the portfolio calculated according to the traditional theory, which shows the characteristics of a single asset structure and risk-free asset allocation. The phenomenon of “limited participation” has attracted academic attention. Several scholars have studied it from different perspectives and found that the background risk of being unable to trade is an important reason that the traditional theory is contrary to the actual situation, and the background risk research for health status is an important part of families' “limited participation” in financial market theory (1, 2).

Compared with developed countries, the phenomenon of “limited participation” in China's financial market is more obvious, and the proportion of risky financial asset allocation in most households is generally low: the participation rate of risky financial assets is only 12.77%, while the participation rate of risk-free financial assets has risen to 74.09%^1^. Which factors cause the phenomenon of “limited participation” of families in the financial market in China, and which factors can explain the heterogeneity differences between urban and rural areas and regions in the allocation of household financial assets? Many scholars have extensively discussed the background that makes risk analysis a key research direction. Therefore, from the perspective of the social security system and insurance economics, this study holds that whether residents allocate urban and rural basic medical insurance may be an important factor affecting family venture financial investment decisions and causing urban, rural, and regional heterogeneity of market participation.

In the process of asset allocation, households are faced with economic shocks from various aspects such as consumption and employment, among which health shocks are one of the major uncertainties faced by Chinese households. Relevant data^2^ show that in 2017, the per capita medical expenses of inpatients in hospitals were ¥ 8,890.7, while the per capita disposable income of the national residents was ¥ 25,974. The risk of medical expenditure is likely to lead families into economic distress, which will undoubtedly have an important impact on household asset allocation. Hence, medical insurance can influence the allocation of family asset through medical expenses.

According to “the Challenge of Population Aging in China,” issued by the CHARLS research team, although China has made great progress in medical insurance coverage^3^, there is also the problem of low coverage of the low-income groups and the elderly living alone^4^. Different classes enjoy different levels of protection, especially when the groups with the more vulnerable economic status and social security levels are exposed to health risks. This phenomenon has been studied from the perspective of intuitionistic behavior, such as the allocation of medical insurance affecting household consumption and investment, revealing that insurance has the greatest influence on the promotion of non-medical consumption expenditure of low-income families, whereas it has little effect on high-income families (3). Participating in medical insurance has considerably increased the possibility and ratio of investing in risky financial assets (4). In contrast, some scholars have studied the influence of health status and social security degree on family investment risk attitude and financial asset allocation from the perspective of health risk, social security degree, and other risk-related background factors to indirectly draw the conclusion that the basic medical insurance system in urban and rural areas should be strengthened to protect groups that are vulnerable to health risks (5). In sum, although there are many studies on families' “limited participation” in the financial market, the literature on the intermediary effect of risk attitude is still rare from the perspective of basic medical insurance allocation.

This study makes four main contributions to the literature. First, we analyse the impact mechanism of urban and rural residents' basic medical insurance on the allocation of family financial assets from the perspective of risk perception. Second, by studying the evolution of the basic medical insurance system and using China Household Finance Survey (CHFS) 2013, 2015, and 2017 panel data, our research draws conclusions and policy suggestions with intense practical significance. Third, we use data about the new rural cooperative medical system (NCMS), urban residents' medical insurance, and other social security systems, which do not only concern the heterogeneity between urban and rural areas, but also naturally divides urban and rural residents' family samples into two groups—with and without medical insurance—through those systems with voluntary insurance characteristics, which makes the research conclusion more reasonable. Fourth, according to the concept of universal health coverage proposed at the 58th World Health Assembly, the realization of “universal health insurance” consists of three stages: lack of financial protection, the intermediate stage of coverage, and universal health insurance. Most developing countries are committed to establishing a fairer and more effective basic health insurance system, Whose financing channel, coverage and subsidy levels have significant similarities, e.g., Thailand proposed “30 baht plan” for universal health coverage in 2001 (6, 7), Mexico in 2003, established the informal employment population in public health insurance coverage to achieve universal health coverage (8, 9). As a result, we use the Heckman two-step model to explore the impact of participation in the Basic Medical Insurance in Urban and rural areas Rural Residents on families' reasonable choice between risk assets and risk-free assets from China perspective to It is enlightening to the research of basic medical security system in developing countries.

The remainder of this paper is organized as follows. Section Institutional Background provides a review of the relevant literature. Section Literature Review presents empirical methods and data sources. Section Methods and Data presents our empirical results. Section Results presents a robustness test. Section Robustness Test concludes the paper and offers policy recommendations.

## Institutional Background

After more than 70 years of reform and development, China has established a basic medical insurance system whose coverage is nearly all urban and rural residents in the country. At present, China's basic medical security system “three vertical lines and three horizontal lines” backbone structure has been clearly shown. The “three vertical lines” refers to the New Rural Cooperative Medical (NRCM), the Urban Residents' Basic Medical Insurance (URBMI) and the Basic Medical insurance for urban and rural residents. These three systems are social insurance systems organized and implemented by the state, and they are also the main parts of the basic medical security system. At the same time, these three systems reside in the middle layer of the “three horizontal lines.” The “bottom horizontal line” of the “horizontal line” is mainly targeted at people in need, which needs to be supplemented by urban and rural medical assistance and social charitable donations. “Top horizontal” is aimed at the people's higher medical needs, which need to be met through supplementary medical insurance and commercial health insurance.

### Introduction to the NRCM

The new rural cooperative medical insurance is a mutual aid system for farmers that is organized, guided and supported by the government, attended voluntarily by farmers, raised by individuals, collectivists and the government, and coordinated by serious diseases as a whole. It adopts the means of individual pay fees, collective support and government funding to raise funds.

In October 2002, China made it clear that governments at all levels should actively guide farmers to establish a new type of rural cooperative medical care system based on overall planning for serious diseases. In 2009, China made an important strategic plan to deepen the reform of the medical and health care system, and established the new agricultural cooperation as the basic medical security system in rural areas. On January 29, 2015, the National Health and Family Planning Commission and the Ministry of Finance issued a notice on doing well the work of the new rural cooperative medical care in 2015, and proposed that the per capita subsidy standard of the new rural cooperative medical care at all levels of finance was increased by 60 yuan based on 2014, reaching 380 yuan.

In 2017, per capita subsidies for the new rural cooperative medical care system from governments at all levels were increased by 30 yuan over 2016 to 450 yuan. The central government will provide subsidies for 80% of the new rural cooperative medical care system in the western region and 60% of the new rural cooperative medical care system in the central region, and a certain proportion of the new rural cooperative medical care system for the provinces in the eastern region. Individual contributions to farmers will be raised by 30 yuan over the 2016 figure, to about 180 yuan on average nationwide in principle. We will explore the establishment of a stable and sustainable financing mechanism that is commensurate with the level of economic and social development and the affordability of all parties.

### Introduction to the URBMI

Since the founding of the People's Republic of China, China's labor insurance and public medical care systems have clearly defined the scope of direct family members supported by state-owned enterprise employees and state staff, and realized the “half-cost” medical insurance system for direct family members, which has largely solved the medical security problem for urban residents. But it is undoubtedly the product of exceptional political and economic times. Since the reform and opening, especially the construction of socialist market economic system, great changes have taken place in China's economic and social environment. With the gradual establishment and improvement of the basic medical insurance system for urban workers since 1998, the medical insurance system for urban workers has realized the transformation from the “state-unit” security system to the “state-society” insurance system. With the continuous changes of medical security reform, the traditional labor insurance system for family members no longer exists, and the original public medical care system also exists in name only. The half-cost medical care system for the relatives and children of urban workers and the public medical care system for college and technical secondary school students account for a small proportion of the population that should be covered.

After the reform, the coverage of medical security is limited and low. According to the Ministry of Health's third national health service survey in 2004, many urban residents are excluded from the institutional protection system. The proportion of urban population enjoying basic medical insurance for urban workers was 30.2 percent. Free medical care 4.0%, labor insurance 4.6%, commercial insurance 5.6%, and no medical insurance 44.8%. However, international experience shows that in the health insurance system, once employees are insured, their immediate family members (spouse and children) who are not working are generally automatically insured. By contrast, this is the biggest institutional defect of the basic medical insurance for urban workers in China.

Therefore, in order to achieve the goal of establishing a medical security system that basically covers all urban and rural residents, the State Council decided to carry out a pilot program of basic medical insurance for urban residents from 2007. The guidance of the State Council on the pilot of basic medical insurance for urban residents requires that the pilot will be launched in 2–3 cities in eligible provinces in 2007, expanded in 2008, and strive to reach more than 80% of the pilot cities in 2009. In 2010, the pilot will be fully rolled out across the country and gradually cover all non-working urban residents. “Since then, the state has issued a series of policy documents on the construction and improvement of basic medical insurance policies for urban residents. Among them, the “Opinions on the Management of Medical Service of the Basic Medical Insurance for Urban Residents” made it clear that the medical service management of the basic medical insurance for urban residents includes the scope of medical service, fixed-point management and the settlement and management of medical expenses. The Ministry of Human Resources and Social Security's notice on the work of basic medical insurance for urban residents in 2008 stipulated that the scope of the pilot will be expanded to 50% of the cities. “In 2008, the government subsidy for insured residents in pilot cities will be raised from no <40 yuan in 2007 to no <80 yuan per person. The central government will provide 40 yuan per person for the central and western regions, and the subsidy for the eastern regions will be raised in parallel with the new rural cooperative medical care system.”

### Implementation of the Integration Policy

In January 2016, the State Council issued the Opinions on the Integration of the Basic Medical Insurance System for Urban and Rural Residents, which combines the basic medical insurance for urban residents and the new rural cooperative medical care system into a unified basic medical insurance for urban and rural residents, covering all urban and rural residents except those who should be covered by the basic medical insurance for employees. At present, except for Tibet, all provinces have made plans and arrangements under the framework of the Opinions or issued policy documents on integrating urban and rural medical insurance. Among them, Tianjin, Chongqing, Ningxia and other provinces had already combined urban and rural medical insurance before the Opinions were issued, and further refined and improved after the Opinions were issued. Whether the first exploration in accordance with local conditions before the issue of the Opinions, or the self-improvement under the top-level design and unified arrangement after the issue of the Opinions, there are some common rules and some local characteristic practices. According to the policy documents of coordinated urban and rural medical insurance in various regions, the contents of the integration process of urban and rural medical insurance systems include:

All localities take “six unifications” as the basic guidance, that is, unified coverage, unified financing policies, unified security treatment, unified medical insurance catalog, unified fixed-point management, and unified white fund management. Individual provinces outside of the “six” and put forward the “unity” seven “eight unified” nine “and so on, including organization of unified management system, management, health supervision, as a whole layer pick, information system and guarantee mechanism of a serious illness, etc.”

In Heilongjiang, Shandong, Chongqing, Shanghai, Tianjin, Hebei and other 22 provinces and the Xinjiang Production and Construction Corps, the basic medical insurance for urban and rural residents has been integrated and handed over to local social departments for unified management. In addition, the medical insurance management of urban and rural residents in Shaanxi Province is centralized to the health department. At the same time, some regions set up medical insurance offices outside the human resources, social security and health departments for overall management, such as Fujian Province. In addition, some provincial-level documents do not specify the specific ownership of medical insurance management, requiring the establishment of coordination groups and other organizations to promote the implementation of medical insurance for urban and rural residents, or to explore their own practices in local cities.

In addition to the proportion of payment in Shanghai, various places according to their actual situation put forward a system of one file, one system of two files or one system of three files and other fixed financing methods, or in the provincial documents did not stipulate specific payment levels, allowing each prefecture-level city to set up their own different levels, with 2–3 years of transition period to achieve the final unification of payment. At the same time, various places put forward to increase the proportion of individual contribution, or clear the lower limit of the proportion of individual contribution in the total amount of financing, such as Fujian Province put forward that the part of individual contribution should not be <25% of the total level of financing in principle.

The general adoption of “treatment is not high on the low, the catalog is not wide on the narrow” principle, emphasizing the treatment and integration before the comparison is not reduced. Overall, after the integration of medical insurance for urban and rural residents, the proportion of hospitalization expenses paid will remain around 75%, and the proportion of outpatient care will be around 50%, so as to narrow the gap with the actual reimbursement rate as much as possible. The specific payment ratio varies according to the level of medical institutions, with a preference for primary medical and health institutions. Some provinces also stipulate the payment ratio difference between different medical institutions.

## Literature Review

Theoretical research on the distribution of household financial assets can be traced back to the portfolio theory advanced by (10), which analyses asset selection under the condition of single-phase perspective and uncertainty, measures asset risk and return by variance and expectation, and concludes that rational investors allocate their assets based on the optimal portfolio of mean, variance, and hedge risk by diversifying their investments. Tobin (11) introduced the concept of risk-free assets based on Markowitz's research and proposed the “two-fund separation theorem.” Based on Tobin and Markowitz's theoretical research, Sharpe (12) further divides portfolio risk into non-decentralized systemic risk and dispersible non-systematic risk and develops the capital asset pricing model (capital asset pricing model). Samuelson and Merton (13) extend the predecessor's proposed single-phase static model over multiple periods to make it more responsive to real investment. According to the traditional investment theory, rational investors choose to allocate part of their funds to risky financial assets, depending on their risk attitude, which is contrary to the reality of the families' “limited participation” in the financial market phenomenon. This phenomenon has invited in-depth study and extensive discussion of the reasons for the deviation between traditional theory and reality, among which the study of background risk is vital.

In addition to the risk of tradable asset price fluctuations, which are mainly studied by the classical investment theory, investors also face non-tradable background risks, including health risks, social security degree, labor income risks, real estate risks, and so on. The first is research on health risks, which has a negative impact on household venture asset investment by making investors more cautious about risk (14); households with poor health are less likely to hold risky financial assets than their healthier counterparts (15, 16) studied the influence of the health status on the allocation of family financial assets from the point of view of the health status of the middle-aged and the elderly, concluding that the health status had a significant negative impact on the proportion of investment real estate holders. However, the impact on liquidity risk assets was not significant, further supporting Edwards (17), who concluded that the health status of the elderly had the most significant impact on the choice of family assets. Some scholars have put forward different views, arguing that the health status of family members and the degree of risk aversion have no significant impact on their venture capital (18–20).

The second concerns the study of the degree of social security. The study of the allocation of family financial assets by the degree of social security can be traced back to (21), who notes that social security has an asset substitution effect and retirement effect. The asset substitution effect refers to the crowding-out effect of social security on family preventive savings, and the retirement effect means that people with social security are more inclined to retire early. Consequently, the influence of the degree of social security on the allocation of household financial assets is the relative size of the effects of asset allocation and retirement. Therefore, before the social security system was highly popularized in China, the social security system showed hierarchical characteristics, in which families with different economic and social statuses enjoyed different conditions of social security, and the impact of social security on family financial asset allocation was mainly reflected in crowding-out residents' consumption and investment (22, 23). With the continuous improvement of the social security system and the gradual improvement of the popularization rate, some scholars who study the factor of social security have come to the opposite conclusion; that is, the social security system can stimulate the growth of household consumption expenditure and risky investment to a certain extent (24). The third relates to the study of labor income risk and real estate risk. Scholars find that the labor income risk of family financiers will make investors more inclined to risk aversion, and have a negative impact on risk asset allocation (25, 26), while real estate risk has two effects on risky financial assets: the crowding-out effect and the asset allocation effect (27–29).

Some research on families' “limited participation” in the financial market are based on other perspectives besides background risk: one of which is that preventive saving has a negative effect on the allocation of risky financial assets by promoting households to accumulate more liquid risk-free assets and reducing financial risk exposure (30). China's family investment presents certain life cycle effect characteristics; however, the performance of different kinds of financial assets is inconsistent (31–33) concluded that the proportion of the elderly population has a positive effect on the holding of risk-free assets, represented by bank savings, and a negative impact on the holding of risk-free assets, represented by stocks, to a certain extent by further subdividing age groups. The above scholars' achievements further improve the research system of families' “limited participation” in the financial market.

The impact of the social security factor on the allocation of family financial assets has been widely studied. Most scholars agree that the allocation of insurance can enhance, to some extent, the depth and breadth of household venture capital investment (34). From the perspective of the endowment insurance, families are motivated to make financial and investment arrangements in their life cycle through savings or endowment insurance, where the latter can replace the former to a certain extent (35). Further research shows that the positive effect of health insurance allocation on family financial asset allocation has a significant impact on highly educated population, but not on their less-educated counterparts (36); moreover, urban and rural basic medical insurance (including new rural cooperative medical insurance, urban workers' medical insurance, etc.) drives urban and rural household consumption (34).

In conclusion, existing research mainly studies the influence of factors such as health status, risk attitude, social security degree, and so on, based on life cycle theory and preventive savings, which has made an important contribution to further enrich the theoretical research on household financial asset allocation. Although there is a certain relationship between health status and basic medical insurance, they have completely different channels of influence on families' choice of financial assets; that is, health status may affect the allocation of family financial assets through medical expenditures, and basic medical insurance may affect the choice of family financial assets through individual risk attitudes. Current research mainly focuses on the impact of health risk factors but less on basic medical insurance, highlighting the practical value of studying basic medical insurance. However, existing literature on basic medical insurance commonly studies a single type of insurance in urban and rural basic medical insurance systems and ignores the details of system evolution. This study examines the influence of basic medical insurance on family financial asset allocation from the perspective of the evolution of the basic medical insurance system by using the micro data of the NCMS, urban residents' medical insurance, and urban and rural residents' basic medical insurance.

## Methods and Data

Microcosmic data at the household level selected in this study are derived from the 2013, 2015, and 2017 issues of the CHFS. We selected the data after 2013 because 2012 was just the tenth anniversary of the urban residents' medical insurance pilot which started in 2002, and the fifth anniversary of the NCMS pilot started in 2007. So far, the implementation of these two medical insurance systems tends to be perfect, which may enhance the representativeness of the research results. Therefore, the CHFS micro data in 2013 and the latter two periods were selected.

We use the Heckman two-step model to investigate the micro information of the allocation of family risk assets and non-risk assets, participation in basic medical insurance, risk attitude, years of education, work conditions, health status, family expenditures, and household registration status. China's household finance database, whose basic characteristics of the survey samples are similar to those of the 2010 national census, has excellent data quality and is widely used by researchers.

### Model Setting

In order to study the impact of the participation of urban and rural basic medical insurance on the family asset allocation, we need to set the core explained variable to whether the family held financial assets and the family held financial assets. However, this order of study is not interchangeable. Because if we first study the proportion of families holding financial assets, then the whole family sample will include families who do not hold financial assets, which will produce endogenous problems, resulting in deviation in the results. Therefore, we should solve the endogenous problem caused by the deviation of sample selection. Specifically, we need to perform a sample correction.

Since Heckman two-step method can well-solve the endogenous problem caused by sample selection deviation. Therefore, this paper chooses it as the main model. The null hypothesis of the model is that participation in medical insurance for urban and rural residents has no impact on the family financial asset allocation. If a small probability event occurs in our regression trial, we doubt the correctness of the null hypothesis. We can then get the influence of medical insurance for urban and rural residents on family financial asset allocation.

The first step of the Heckman two-step method is to select the equation, in which we estimate the impact of participating in urban and rural residents' medical insurance on whether families hold financial assets, and simultaneously obtain the inverse Mills ratio λ estimated by a probit model. The second step concerns the quantitative equation, in which the inverse Mills ratio λ obtained from Equation (1) is substituted into the quantity equation as the control variable to ensure that the sample households in this step are all households holding financial assets, as follows:

(1) $$\begin{array}{r} pro\left\{Y_{it}=1\right\}=\alpha_{0}+\alpha_{1}P\text{u}\_ins_{it}+\alpha_{2}X_{it}+\phi_{t}+\rho_{p}+\mu_{it} \end{array}$$

(2) $$\begin{array}{r} \ln\;asset_{it}=\alpha_{0}+\alpha_{1}P\text{u}\_ins_{it}+\alpha_{2}X_{it}+\phi_{t}+\rho_{p}+\mu_{it} \end{array}$$

*i* in the equation represents the family, and t represents the period. The explained variable *Y*_*it*_ in Equation (1) is a virtual variable as to whether family *i* holds risky or risk-free assets in period *t*. The explained variable in Equation (2) measures the proportion of financial assets held by households. The first term α_0_ on the right is a constant term. The second *Pu* < *uscore* > *ins*_*it*_ is the core explanatory variable, indicating whether to participate in medical insurance for urban and rural residents, medical insurance for urban residents, or NCMS in different regressions. The third term *X*_*it*_ represents the control variables at the individual and household levels, including years of education, work status, health status, family expenditure, household status, and so on. The fourth term is the time-control variable. The fifth term ρ_*p*_ reflects the fixed effects of the province. The last term μ_*it*_ means residual, which is assumed to be distributed independently, identically, and normally.

### Description of Variables

1. The allocation of financial assets can be measured from four aspects: (1) whether the sample households hold risky assets, (2) the proportion of risky assets held, (3) whether they hold riskless assets, and (4) the proportion of riskless assets held. Risky assets are composed of stocks, loans, funds, derivatives, Internet financial management, financial management, non-RMB assets, gold, and other risky assets. Risk-free assets are composed of cash, bonds, demand deposits, and time deposits. Consequently, we select whether to hold risky assets or riskless assets, and the corresponding holding ratio as the core explained variable.

2. Participation in basic medical insurance for urban and rural areas. The State Council's opinions on integrating the basic medical insurance system of urban and rural residents issued on 12 January 2016 integrated the medical insurance of urban residents with the new rural integration into urban and rural residents' medical insurance. Consequently, we extract from 2013 to 2015 CHFS microdata the two variables of whether to participate in urban residents' medical insurance and whether to participate in the new rural cooperative. If one of the two values is 1, then the virtual variable “whether to participate in urban and rural residents” medical insurance' is assigned a value of 1. Additionally, we extracted from the 2017 CHFS microdata the three variables of whether to participate in urban residents' medical insurance, whether to participate in NCMS, and whether to participate in urban and rural residents' medical insurance as the core explanatory variables.

3. Risk attitude, which affects the probability and proportion of households holding financial assets, is selected as the intermediary variable to test the intermediary effect. We use the answer to the following question in the CHFS survey questionnaire: “Suppose there are two lottery tickets for you to choose from; if you choose the first one, you have a 100% chance to get 4,000 yuan; if you choose the second one, you have a 50% chance to get 10,000 yuan and 50% chance to get nothing. Which one would you like to choose as an indicator of risk attitude?” The risk attitude index of the families who choose “the first one” is 1, and that of the families who choose “the second one” is 0. In this way, risk attitude = 0 indicates risk preference, and 1 means risk aversion.

4. Other control variables: demographic variables and family economic situation will also affect the allocation of family financial assets; thus, the demographic variables selected in this study include age, sex, marital status, resident education level, and family structure (including the total family population and whether there are children). In addition, given that China has a vast territory and there are great cultural and economic differences among provinces, we also control for the fixed effect of provinces.

### Description of Data Validity Processing

Considering the multi-selection of CHFS questionnaire data used in this study and different annual policies, we address the original data as follows:

1. For family data: only tracking samples from 2015 to 2017 data starting in 2013 are retained, and non-tracking samples are deleted.
2. For the variable of whether to participate in urban and rural residents' medical insurance: In 2016, the State Council put forward “opinions on integrating the basic medical insurance system of urban and rural residents,” requiring that urban and rural residents' medical insurance should cover all urban residents' medical insurance and all new rural residents. Therefore, the variable of “participating in urban and rural residents” medical insurance' in this paper is integrated in the following ways. For the question, “which medical insurance do you currently have?” in the 2013 CHFS questionnaire, we combined the data of “basic medical insurance for urban residents” and “new rural cooperative medical insurance” as “participating in medical insurance for urban and rural residents.” This is because “vigorously promoting medical insurance for urban and rural residents” was proposed by the State Council on May 18, 2013, and then there is no option of “Basic Medical Insurance for Urban and Rural Residents” in the 2013 questionnaire. In the questionnaire for 2015 and 2017, we combine the data of “basic medical insurance for urban residents,” “new rural cooperative medical insurance,” and “Basic Medical Insurance for Urban and Rural Residents” as “participating in medical insurance for urban and rural residents.”
3. For family size variable: if the respondent is still one of the family members, then we add one to the number of family members other than the respondent and record it as “family number.”
4. For education level variable, we merge “primary school” and “junior high school” and record them as “primary or junior high school diploma.” We merge “high school,” “technical secondary school/vocational high school” and “college/higher vocational education” and record them as “high school or college.” We merge “undergraduate degree,” “master's degree,” and “doctoral degree” and record them as “undergraduate degree or above.”
5. For household asset variables, “liquidity assets,” “fixed assets,” and “financial investment” are merged and recorded as “household assets.”
6. For risk attitude variables: In the 2013, 2015, and 2017 CHFS questionnaire, we use the answer to the lottery question above as an indicator of risk attitude. The risk attitude of the families who choose “the first one” is recorded as risk aversion and the opposite is risk preference.
7. For missing values, if the variable data of current assets, fixed assets, and financial investment variables are missing, the corresponding value is 0. If the sex and age of the sample are missing and the household income is negative, then there are missing answers or obvious logical errors in these questionnaires, and we delete them.

Table 1 is the descriptive statistical results of all variables which undergo descriptive regression after selection and cleaning.

**Table 1:** Descriptive statistics of variables.

**Variable type**	**Variable name**	**Variable symbol**	**Mean value**	**Standard deviation**	**Minimum value**	**Maximum value**
Explained variables	Whether to hold risky assets (hdra, yes = 1, no = 0)	Risk_take	0.19	0.39	0	1
	Whether to hold risk-free assets (hdrfa, yes = 1, no = 0)	Lasset_take	0.95	0.21	0	1
	Proportion of risky assets (propra, risky assets/total assets)	Risk_ratio	0.05	0.15	0	1
	Proportion of risk-free assets (proprfa, risk-free assets/total assets)	Lasset_ratio	0.39	0.43	0	1
Explanatory variables	Medical insurance for urban residents (hdins-urban, yes = 1, no = 0)	Urban_ins	0.13	0.34	0	1
	NCMS (hdins-rural, yes = 1, no = 0)	Rural_ins	0.49	0.5	0	1
	Basic Medical Insurance for Urban and Rural Residents (hdins, yes = 1, no = 0)	UrRural_ins	0.58	0.49	0	1
Intermediary variable	Risk attitude^a^ (rat)	Risk_atti	2.64	0.57	1	3
Individual level control variables	Sex (M = 1, F = 0)	Sex	0.78	0.41	0	1
	Age (years)	Age	53.14	13.89	3	113
	Level of education (reference group is not in school)	Edu				
	Primary or junior high school education (pri-jun edu)	Edu1	0.57	0.5	0	1
	High school or college (hi-co edu)	Edu2	0.27	0.45	0	1
	Bachelor's degree or above (ba edu)	Edu3	0.08	0.27	0	1
	Years of education (y-edu)	Eduyear	9.09	4	0	23
	Local account (la, yes = 1, no = 0)	Hukou	0.63	0.48	0	1
	Marital status (base group unmarried)	Marr				
	Married (m)	Marr1	0.87	0.33	0	1
	Divorce or widowhood (d-w)	Marr2	0.1	0.29	0	1
	Brothers and sisters (bro-sis)	Siblings	1.57	2.04	0	16
	Working conditions (wco, yes = 1, no = 0)	Work	0.67	0.47	0	1
	Self-assessment of health status (hs, 1–5, healthy-unhealthy)	Health	2.97	1.12	1	5
Family level control variables	Whether to hold the commercial health insurance (pri-hi)	pr_ins	0.033	0.179	0	1
	Family income (in)	Hh_income	67,686	83,123	0	550,440
	Income logarithm (in-log)	Ln_hh_income	10.31	1.88	−1.74	13.22
	Family assets (as)	L_asset	38,451	83,428	0	530,000
	Asset logarithm (as-log)	Lnasset	10.95	2.88	0	15.67
	Household liabilities (hl)	Hh_debt	34,992	114,305	0	797,537
	Debt logarithm (de-log)	Lndebt	2.84	4.8	−4.2	13.59
	Medical expenditure (me)	Med_exp	2,664	13,474	0	1.5 × 10^6^
	Logarithm of medical expenditure (me-log)	Lnmed_exp	2.86	3.78	−0.69	14.22
	Number of families (fn)	Fam_num	3.58	1.75	1	11
	Average family years of education (fay-edu)	a_edu	8.141	3.762	0	23
	Average family age (fa-ag)	A_age	47.17	15.54	4	113
	The proportion of men in the family (fpropm)	R_man	0.5	0.2	0	1
	Proportion of households receiving pensions(fpropp)	R_pension	0.25	0.38	0	1
	The proportion of working persons in the family (fpropw)	R_job	0.42	0.42	0	1
	Family dependency ratio (fpropr)	R_raise	0.25	0.34	0	1

a *Risk attitude measured by the willingness to invest after winning the lottery; risk aversion = 1, risk preference = 0*.

## Results

### Benchmark Empirical Model

Table 2 reports the benchmark regression results of the Heckman two-step model. During the experiment process, we add the time dummy variable and province variable for the core control variables in the regression. Column (1) indicates that the impact of participation in urban and rural residents' medical insurance on whether households hold risky assets is significant at the 1% confidence level. Households with urban and rural residents' medical insurance are 12.1% less likely to hold risky assets than those without medical insurance, which indicates that the allocation of medical insurance for urban and rural residents has a significant negative impact on household risky financial assets, whereas the allocation of medical insurance for urban residents and NCMS have a positive but less significant impact. In Column (2), the effect of holding urban and rural residents' medical insurance on the proportion of risk assets held by families is significant at the 1% confidence level; that is, holding urban and rural residents' medical insurance has a certain impact on the decline of the proportion of household risky assets. Further, the impact of the allocation of urban residents' medical insurance and NCMS is similar. Columns (3) and (4) reveal the regression results from the perspective of risk-free assets; the allocation of urban and rural basic medical insurance has a certain positive impact on the probability of family holding risk-free assets but has a certain negative impact on the proportion. Therefore, at present, urban and rural basic medical insurance has a certain asset substitution effect on household financial investment and preventive savings, which is consistent with the theory put forward by (21).

**Table 2:** Benchmark regression.

**Explanatory variable**	**Risk assets/Risk-free assets**		**Risk assets/Risk-free assets**		**Risk assets/Risk-free assets**	
	**hdra**	**propra**	**hdra**	**propra**	**hdra**	**propra**
	**(1)**	**(2)**	**(3)**	**(4)**	**(5)**	**(6)**
**Part 1: Risk assets**						
hdins	−0.1210^***^ (0.0244)	−0.0149^***^ (0.0019)				
hdins urban			0.0252 (0.0305)	−0.0012 (0.0024)		
hdins-rural					0.0005 (0.0594)	−0.0040 (0.0025)
Pri-hi	0.6070^***^ (0.0439)	0.0402^***^ (0.0056)	0.6520^***^ (0.0489)	0.0298^***^ (0.0065)	0.3570^***^ (0.108)	0.0499^***^ (0.0088)
Bro-sis	0.0138^**^ (0.0067)	0.0004 (0.0004)	0.0100 (0.0071)	−0.0003 (0.0006)	0.0201 (0.0126)	0.0025^***^ (0.0006)
gender	0.0066 (0.0278)	−0.0013 (0.0022)	0.0192 (0.0303)	−0.0007 (0.0028)	−0.0089 (0.0747)	−0.0024 (0.0028)
age	−0.0087^***^ (0.0015)	−0.0005^***^ (0.0001)	−0.0062^***^ (0.0016)	−0.0001 (0.0002)	−0.0109^***^ (0.0027)	−0.0017^***^ (0.0002)
Pri-jun edu	−0.1160 (0.0866)	−0.0243^***^ (0.0041)	−0.1870 (0.1170)	−0.0340^***^ (0.0065)	0.0082 (0.1350)	−0.0024 (0.0042)
Hi-co edu	−0.0347 (0.1150)	−0.0164^**^ (0.0064)	−0.0918 (0.1480)	−0.0313^***^ (0.0096)	−0.0167 (0.1940)	−0.0036 (0.0071)
Ba edu	−0.1080 (0.1450)	0.0007 (0.0092)	−0.1990 (0.1810)	−0.0175 (0.0131)	−0.1310 (0.4380)	0.0376 (0.0421)
Y-edu	0.0426^***^ (0.0089)	0.0036^***^ (0.0007)	0.0561^***^ (0.0107)	0.0039^***^ (0.0010)	0.0208 (0.0158)	0.0030^***^ (0.0007)
La	0.0611^***^ (0.0218)	0.0172^***^ (0.0020)	0.0464^*^ (0.0261)	0.0103^***^ (0.0025)	0.0806 (0.0690)	0.0155^***^ (0.0038)
M	0.1030^*^ (0.0598)	−0.0011 (0.0051)	0.1440^**^ (0.0648)	−0.0018 (0.0064)	−0.1970 (0.1510)	−0.0318^***^ (0.0078)
D-w	0.1040 (0.0714)	−0.0026 (0.0056)	0.1170 (0.0784)	−0.0057 (0.0072)	−0.1090 (0.1720)	−0.0176^**^ (0.0074)
Wco	0.0847^***^ (0.0315)	−0.0021 (0.0020)	0.0782^**^ (0.0370)	−0.0054^*^ (0.0029)	0.1660^**^ (0.0648)	0.0215^***^ (0.0035)
Hs	−0.0477^***^ (0.0100)	−0.0008 (0.0007)	−0.0329^***^ (0.0118)	0.0001 (0.0010)	−0.0790^***^ (0.0185)	−0.0101^***^ (0.0014)
Me-log	0.0056^*^ (0.0032)	0.0001 (0.0002)	0.0051 (0.0037)	−0.0001 (0.0003)	0.0101 (0.0071)	0.00126^***^ (0.0003)
Me	−0.0001 (0.0001)	−0.0001 (0.0001)	−0.0001 (0.0001)	−0.0001 (0.0001)	−0.0001 (0.0001)	−0.0001^***^ (0.0001)
In-log	0.1270^***^ (0.0095)	0.0046^***^ (0.0011)	0.1230^***^ (0.0107)	0.0009 (0.0013)	0.1350^***^ (0.0203)	0.0188^***^ (0.0025)
As-log	0.2880^***^ (0.0063)	0.0070^***^ (0.0025)	0.2800^***^ (0.0072)	−0.0022 (0.0028)	0.3010^***^ (0.0135)	0.0399^***^ (0.0056)
Fn	−0.0575^***^ (0.0075)	−0.0046^***^ (0.0006)	−0.0648^***^ (0.0092)	−0.0042^***^ (0.0009)	−0.0344^**^ (0.0134)	−0.0058^***^ (0.0008)
Fa-ag	−0.0109^***^ (0.0014)	−0.0004^***^ (0.0001)	−0.0117^***^ (0.0016)	−0.0001 (0.0002)	−0.0072^***^ (0.0026)	−0.0010^***^ (0.0002)
Fpropm	0.0624 (0.0564)	0.0013 (0.0040)	0.0533 (0.0632)	−0.0037 (0.0055)	0.1100 (0.1250)	0.0194^***^ (0.0047)
Fpropp	0.0624 (0.0564)	0.0013 (0.0041)	0.0533 (0.0632)	−0.0037 (0.0055)	0.1100 (0.1250)	0.0194^***^ (0.0047)
Fpropw	−0.1970^***^ (0.0399)	−0.0010 (0.0029)	−0.1880^***^ (0.0464)	0.0031 (0.0039)	−0.1380 (0.0933)	−0.0159^***^ (0.0034)
Fpropr	−0.0486 (0.0416)	−0.0034 (0.0027)	−0.0844^*^ (0.0479)	−0.0043 (0.0039)	0.0581 (0.0944)	0.0132^***^ (0.0026)
Fay-edu	0.0415^***^ (0.0049)	0.0005 (0.0005)	0.0392^***^ (0.0058)	−0.0007 (0.0006)	0.0384^***^ (0.0101)	0.0050^***^ (0.0008)
De-log	−0.0101^***^ (0.0020)	−0.0012^***^ (0.0002)	−0.0061^***^ (0.0023)	−0.0011^***^ (0.0002)	−0.0213^***^ (0.0041)	−0.0031^***^ (0.0004)
**Part 2: Risk-free assets**						
hdins	0.0189 (0.0294)	−0.0352^***^ (0.00323)				
hdins urban			−0.0094 (0.0414)	0.0003 (0.0040)		
hdins-rural					0.1560^***^ (0.0506)	−0.0494^***^ (0.0076)
Pri-hi	0.0727 (0.0827)	0.0215^***^ (0.0057)	0.0075 (0.0909)	0.0257^***^ (0.0061)	0.2190 (0.1710)	0.0332^**^ (0.0157)
Bro-sis	−0.0150^**^ (0.0073)	0.0009 (0.0009)	−0.0134 (0.0105)	0.0005 (0.0010)	−0.0134 (0.0102)	0.0017 (0.0015)
gender	0.0066 (0.0278)	−0.0013 (0.0022)	0.0192 (0.0303)	−0.0007 (0.0028)	−0.0089 (0.0747)	−0.0024 (0.0028)
age	−0.0053^***^ (0.0017)	0.0009^***^ (0.0002)	−0.0046^*^ (0.0025)	0.0013^***^ (0.0002)	−0.0049^**^ (0.0022)	0.0009^***^ (0.0003)
Pri-jun edu	−0.0244 (0.0745)	−0.0746^***^ (0.0097)	0.0455 (0.114)	−0.1090^***^ (0.0139)	−0.0142 (0.1020)	−0.0665^***^ (0.0147)
Hi-co edu	−0.1390 (0.1150)	−0.0576^***^ (0.0136)	−0.0926 (0.1640)	−0.0758^***^ (0.0179)	−0.0930 (0.1660)	−0.0782^***^ (0.0225)
Ba edu	−0.3820^**^ (0.1590)	−0.0362^**^ (0.0174)	−0.3370 (0.2200)	−0.0336 (0.0221)	−0.0447 (0.4010)	−0.1070^**^ (0.0481)
Y-edu	0.0273^***^ (0.0099)	0.0001 (0.0011)	0.0287^**^ (0.0142)	−0.0012 (0.0014)	0.0184 (0.0138)	0.0049^***^ (0.0019)
La	−0.1270^***^ (0.0296)	0.0123^***^ (0.0031)	−0.0950^**^ (0.0380)	0.0132^***^ (0.0038)	−0.1910^***^ (0.0657)	0.0273^***^ (0.0090)
M	0.0980 (0.0700)	−0.0150^*^ (0.0084)	0.0969 (0.0933)	−0.0122 (0.0091)	0.0838 (0.1060)	−0.0288 (0.0195)
D-w	0.0794 (0.0781)	−0.0301^***^ (0.0097)	0.0774 (0.1040)	−0.0289^***^ (0.0107)	0.0505 (0.1180)	−0.0335 (0.0213)
Wco	0.0130 (0.0346)	−0.0152^***^ (0.0040)	0.0984^*^ (0.0510)	−0.0321^***^ (0.0049)	−0.0637 (0.0488)	−0.0011 (0.0073)
Hs	−0.0196^*^ (0.0116)	−0.0081^***^ (0.0013)	−0.0145 (0.0165)	−0.0051^***^ (0.0015)	−0.0174 (0.0161)	−0.0116^***^ (0.0023)
Me-log	0.0092^**^ (0.0038)	−0.0019^***^ (0.0004)	0.0087 (0.0054)	−0.0022^***^ (0.0005)	0.0105^*^ (0.0057)	−0.0019^**^ (0.0009)
Me	−0.0001^***^ (0.0001)	0.0001^***^ (0.0001)	−0.0001 (0.0001)	0.0001^***^ (0.0001)	−0.0001^**^ (0.0001)	0.0001^**^ (0.0001)
In-log	0.0380^***^ (0.0056)	0.0027^***^ (0.0009)	0.0273^***^ (0.0077)	0.0028^***^ (0.0010)	0.0510^***^ (0.0087)	0.0022 (0.0016)
As-log	0.1780^***^ (0.0049)	−0.1410^***^ (0.0011)	0.1580^***^ (0.0069)	−0.1570^***^ (0.0014)	0.1990^***^ (0.0072)	−0.1190^***^ (0.0022)
Fn	−0.0247^***^ (0.0071)	0.0010 (0.0009)	−0.0248^**^ (0.0112)	0.0024^**^ (0.0012)	−0.0156^*^ (0.0094)	−0.0015 (0.0014)
Fa-ag	−0.0003 (0.0014)	−0.0001 (0.0002)	−0.0022 (0.0021)	0.0006^***^ (0.0002)	0.0029 (0.0020)	−0.0005^*^ (0.0003)
Fpropm	−0.1360^**^ (0.0630)	0.0135^*^ (0.0074)	−0.1330 (0.0840)	0.0223^***^ (0.0086)	−0.1150 (0.0954)	−0.0018 (0.0140)
Fpropp	0.0656 (0.0414)	−0.0169^***^ (0.0052)	0.1180^**^ (0.0590)	−0.0440^***^ (0.0062)	0.0128 (0.0588)	0.0130 (0.0094)
Fpropw	−0.0108 (0.0475)	−0.0046 (0.0053)	−0.0091 (0.0693)	0.0072 (0.0063)	0.0756 (0.0695)	0.0047 (0.0106)
Fpropr	0.0717 (0.0440)	0.0116^**^ (0.0052)	0.0917 (0.0625)	0.0091 (0.0063)	0.0553 (0.0623)	−0.0018 (0.0093)
Fay-edu	0.0205^***^ (0.0059)	0.00147^**^ (0.0007)	0.0130 (0.0085)	0.0010 (0.0008)	0.0207^**^ (0.0085)	0.0001 (0.0012)
De-log	−0.0294^***^ (0.0025)	−0.0009^***^ (0.0003)	−0.0231^***^ (0.0035)	−0.0012^***^ (0.0003)	−0.0353^***^ (0.0035)	−0.0001 (0.0005)

*Standard errors in parentheses*.

* *p < 0.1,*

** *p < 0.05,*

*** *p < 0.01*.

### Intermediary Effect Test

The practice of allocating basic medical insurance in urban and rural areas may increase residents' perception of risk, make families more cautious in allocating risky financial assets, and thus reduce the probability and proportion of holding risky assets such as stocks (37, 38). Therefore, based on the benchmark regression model, we further explore the intermediate variables of the impact of urban and rural basic medical insurance participation on the allocation of household financial assets. To test the intermediary effect, we set whether families hold risk assets and non-risk assets and the corresponding holding ratio as the core explanatory variable; risk attitude as an intermediary variable; and whether they participate in urban and rural residents' medical insurance, whether they participate in urban and rural residents' medical insurance, and whether they participate in NCMS as the core explanatory variables.

As shown in Column (1) of Table 3, in urban and rural basic medical insurance, the allocation of urban and rural residents' medical insurance and NCMS will increase the degree of risk aversion, which can be decreased by the allocation of urban residents' medical insurance, and also explains the results of the benchmark regression model to a certain extent.

**Table 3:** Intermediary effect regression (risk assets).

**Explanatory variable**		**Risk assets**				
		**(1)**	**(2)**	**(3)**	**(4)**	**(5)**
		**Impact on rats**	**Intermediate effect**	**Direct effect**	**Intermediate effect**	**Direct effect**
			**The impact of rat on hdra**	**Fixed rat depends on hdra**	**The influence of rat on the propra**	**Fixed rat to look at the propra**
**Part 1: Medical insurance for urban and rural residents**						
	hdins	0.0217 (0.0195)		−0.1200*** (0.0245)		−0.0150*** (0.0019)
	Rat		−0.1200*** (0.0248)	−0.1200*** (0.0248)	−0.0101*** (0.00222)	−0.0099*** (0.0022)
Pri-hi		−0.0937** (0.0470)	0.6040*** (0.0439)	0.6040*** (0.0439)	0.0404*** (0.0057)	0.03990*** (0.0057)
Bro-sis		0.0131*** (0.0045)	0.0149** (0.0062)	0.0151** (0.0062)	0.0004 (0.0004)	0.0005 (0.0004)
gender		−0.1260*** (0.0230)	−0.0044 (0.0279)	0.0004 (0.0279)	−0.0027 (0.0022)	−0.0021 (0.0022)
age		0.0041*** (0.0013)	−0.0080*** (0.0015)	−0.0082*** (0.0014)	−0.0004*** (0.0001)	−0.0005*** (0.0001)
Pri-jun edu		0.0001 (0.0572)	−0.1210 (0.0873)	−0.0986 (0.0872)	−0.0261*** (0.0041)	−0.0230*** (0.0041)
Hi-co edu		−0.1010 (0.0818)	−0.0292 (0.1160)	−0.0168 (0.1160)	−0.0161** (0.0065)	−0.0145** (0.0065)
Ba edu		−0.0999 (0.1060)	−0.1010 (0.1460)	−0.0915 (0.1450)	0.0022 (0.0093)	0.0031 (0.0093)
Y-edu		0.0097 (0.0069)	0.0458*** (0.0089)	0.0422*** (0.0089)	0.0039*** (0.0007)	0.0035*** (0.0007)
La		−0.0162 (0.0209)	0.0572*** (0.0219)	0.0677*** (0.0221)	0.0163*** (0.0020)	0.0178*** (0.0020)
M		0.1550*** (0.0417)	0.1010* (0.0604)	0.0997* (0.0603)	−0.0011 (0.0052)	−0.0009 (0.0052)
D-w		0.0781 (0.0501)	0.0992 (0.0719)	0.0965 (0.0718)	−0.0027 (0.0057)	−0.0027 (0.0056)
Wco		0.0070 (0.0307)	0.0798** (0.0315)	0.0856*** (0.0316)	−0.0028 (0.0020)	−0.0019 (0.0020)
Hs		0.0314*** (0.0077)	−0.0475*** (0.0100)	−0.0476*** (0.0100)	−0.0010 (0.0007)	−0.0009 (0.0007)
Me-log		0.0015 (0.0027)	0.0062* (0.0033)	0.0058* (0.0033)	0.0001 (0.0002)	0.0001 (0.0002)
Me		−0.0001 (0.0001)	−0.0001 (0.0001)	−0.0001 (0.0001)	−0.0001 (0.0001)	−0.0001 (0.0001)
In-log		−0.0087** (0.0044)	0.1290*** (0.0095)	0.1250*** (0.0094)	0.0052*** (0.0012)	0.0047*** (0.0011)
As-log		−0.0137*** (0.0031)	0.2890*** (0.0064)	0.2870*** (0.0064)	0.0075*** (0.0025)	0.0069*** (0.0025)
Fn		0.0048 (0.0052)	−0.0608*** (0.0075)	−0.0563*** (0.0076)	−0.0051*** (0.0007)	−0.0045*** (0.0006)
Fa-ag		0.0055*** (0.0012)	−0.0111*** (0.0014)	−0.0108*** (0.0014)	−0.0004*** (0.0001)	−0.0004*** (0.0001)
Fpropm		−0.1600*** (0.0439)	0.0603 (0.0569)	0.0650 (0.0568)	0.0015 (0.0041)	0.0019 (0.0041)
Fpropp		0.0137 (0.0294)	0.1070*** (0.0402)	0.0926** (0.0402)	0.0148*** (0.0023)	0.0137*** (0.0023)
Fpropw		−0.0794** (0.0361)	−0.2200*** (0.0400)	−0.2040*** (0.0401)	−0.0043 (0.0030)	−0.0015 (0.0030)
Fpropr		0.0331 (0.0332)	−0.0364 (0.0418)	−0.0435 (0.0418)	−0.0032 (0.0026)	−0.0039 (0.0027)
**Part 1: Medical insurance for urban and rural residents**						
Fay–edu		−0.0082** (0.0041)	0.0425*** (0.0050)	0.0402*** (0.0050)	0.0009* (0.0005)	0.0005 (0.0005)
De-log		−0.0024 (0.0016)	−0.0104*** (0.0020)	−0.0104*** (0.0020)	−0.00125*** (0.0002)	−0.0012*** (0.0002)
**Part 2: Medical insurance for urban residents**						
	hdins-urban	−0.0459 (0.0300)		0.0187 (0.0307)		−0.0010 (0.0024)
	Rat		−0.1190*** (0.0280)	−0.1180*** (0.0281)	−0.0096*** (0.0029)	−0.0096*** (0.0029)
Pri-hi		−0.0753 (0.0514)	0.6520*** (0.0490)	0.6520*** (0.0490)	0.0300*** (0.0065)	0.0300*** (0.0066)
Bro-sis		0.0231*** (0.0059)	0.0108 (0.0072)	0.0108 (0.0072)	−0.0002 (0.0006)	−0.0002 (0.0006)
gender		−0.1590*** (0.0266)	0.0127 (0.0305)	0.0131 (0.0305)	−0.0014 (0.0029)	−0.0015 (0.0028)
age		0.0043*** (0.0016)	−0.0055*** (0.0018)	−0.0055*** (0.0018)	−0.0001 (0.0001)	−0.0001 (0.0002)
Pri-jun edu		0.0271 (0.0837)	−0.1590 (0.1180)	−0.1600 (0.1180)	−0.0336*** (0.0065)	−0.0335*** (0.0065)
Hi-co edu		−0.0673 (0.1120)	−0.0590 (0.1490)	−0.0603 (0.1490)	−0.0302*** (0.0097)	−0.0301*** (0.0097)
Ba edu		−0.0439 (0.1410)	−0.1610 (0.1820)	−0.1630 (0.1820)	−0.0156 (0.0131)	−0.0156 (0.0131)
Y-edu		0.0071 (0.0090)	0.0542*** (0.0108)	0.0546*** (0.0108)	0.0039*** (0.0010)	0.0039*** (0.0010)
La		−0.0169 (0.0240)	0.0543** (0.0258)	0.0511* (0.0264)	0.0102*** (0.0025)	0.0104*** (0.0025)
M		0.1480*** (0.0485)	0.1340** (0.0652)	0.1340** (0.0652)	−0.0018 (0.0065)	−0.0018 (0.0065)
D-w		0.0428 (0.0597)	0.106 (0.0788)	0.106 (0.0789)	−0.00592 (0.00722)	−0.00594 (0.00722)
Wco		−0.00431 (0.0380)	0.0783** (0.0371)	0.0783** (0.0371)	−0.00508* (0.00289)	−0.00508* (0.00289)
Hs		0.0408*** (0.0097)	−0.0335*** (0.0118)	−0.0334*** (0.0118)	0.0001 (0.0010)	0.0001 (0.0010)
Me-log		0.00191 (0.0034)	0.0053 (0.0037)	0.0054 (0.0037)	−0.0001 (0.0003)	−0.0001 (0.0003)
Me		0.0001 (0.0001)	−0.0001 (0.0001)	−0.0001 (0.0001)	−0.0001 (0.0001)	−0.0001 (0.0001)
In-log		−0.0061 (0.0053)	0.1200*** (0.0106)	0.1210*** (0.0106)	0.0011 (0.0013)	0.0011 (0.0013)
As-log		−0.0170*** (0.0041)	0.2780*** (0.0072)	0.2780*** (0.0072)	−0.0021 (0.0029)	−0.0021 (0.0029)
Fn		0.0183** (0.0074)	−0.0614*** (0.0092)	−0.0615*** (0.0092)	−0.0042*** (0.0009)	−0.0042*** (0.0009)
Fa-ag		0.0060*** (0.0015)	−0.0117*** (0.0016)	−0.0117*** (0.0016)	−0.0001 (0.0001)	−0.0001 (0.0001)
Fpropm		−0.1420*** (0.0526)	0.0560 (0.0636)	0.0559 (0.0636)	−0.0030 (0.0055)	−0.0030 (0.0055)
Fpropp		0.0222 (0.0378)	0.1250*** (0.0462)	0.1270*** (0.0462)	0.0108*** (0.0034)	0.0107*** (0.0034)
Fpropw		−0.0744 (0.0463)	−0.1970*** (0.0466)	−0.1960*** (0.0467)	0.0024 (0.0039)	0.0024 (0.0039)
Fpropr		0.0653 (0.0425)	−0.0786 (0.0482)	−0.0785 (0.0482)	−0.0050 (0.0039)	−0.0050 (0.0039)
Fay-edu		−0.0088 (0.0054)	0.0382*** (0.0059)	0.0382*** (0.0059)	−0.0007 (0.0006)	−0.0007 (0.0006)
De-log		−0.0024 (0.0020)	−0.0064*** (0.0023)	−0.0065*** (0.0023)	−0.0012*** (0.0002)	−0.0012*** (0.0002)
**Part 3: New rural cooperative medical**						
	hdins-rural	0.0506 (0.0418)		0.0130 (0.0596)		−0.0025 (0.0026)
	Rat		−0.1360** (0.0539)	−0.1370** (0.0539)	−0.0180*** (0.0034)	−0.0179*** (0.0034)
Pri-hi		−0.1980 (0.1280)	0.3430*** (0.1070)	0.3430*** (0.1070)	0.0470*** (0.0085)	0.0467*** (0.0085)
Bro-sis		−0.0048 (0.0069)	0.0213* (0.0127)	0.0213* (0.0127)	0.0027*** (0.0006)	0.0027*** (0.0006)
gender		−0.0027 (0.0492)	−0.0184 (0.0747)	−0.0188 (0.0746)	−0.0041 (0.0028)	−0.0040 (0.0028)
age		0.0027 (0.0019)	−0.0111*** (0.0027)	−0.0111*** (0.0027)	−0.0018*** (0.0002)	−0.0018*** (0.0002)
Pri-jun edu		−0.0539 (0.0823)	0.0017 (0.1360)	0.0012 (0.1360)	−0.0025 (0.0042)	−0.0024 (0.0042)
Hi-co edu		−0.1300 (0.1280)	−0.0251 (0.1950)	−0.0249 (0.1950)	−0.0034 (0.0070)	−0.0034 (0.0070)
Ba edu		−0.3960 (0.2920)	−0.1420 (0.4410)	−0.1370 (0.4410)	0.0401 (0.0429)	0.0393 (0.0429)
Y-edu		0.0113 (0.0110)	0.0225 (0.0158)	0.0226 (0.0158)	0.0032*** (0.0007)	0.0032*** (0.0007)
La		−0.0670 (0.0517)	0.0829 (0.0697)	0.0819 (0.0700)	0.0162*** (0.0039)	0.0163*** (0.0039)
M		0.0507 (0.0939)	−0.1720 (0.1530)	−0.1720 (0.1530)	−0.0284*** (0.0077)	−0.0280*** (0.0077)
D-w		0.0667 (0.1040)	−0.0978 (0.1740)	−0.0985 (0.1740)	−0.0160** (0.0074)	−0.0157** (0.0074)
Wco		0.0607 (0.0529)	0.1800*** (0.0647)	0.1790*** (0.0648)	0.0234*** (0.0037)	0.0233*** (0.0037)
Hs		0.0196 (0.0126)	−0.0767*** (0.0186)	−0.0767*** (0.0186)	−0.0099*** (0.0014)	−0.0098*** (0.0014)
Me-log		−0.0005 (0.0044)	0.0106 (0.0071)	0.0106 (0.0071)	0.0014*** (0.0003)	0.0014*** (0.0003)
Me		−0.0001** (0.0001)	−0.0001 (0.0001)	−0.0001 (0.0001)	−0.0001*** (0.0001)	−0.0001*** (0.0001)
In-log		−0.0214*** (0.0081)	0.1340*** (0.0204)	0.1340*** (0.0204)	0.0188*** (0.0025)	0.0187*** (0.0025)
As-log		−0.0121** (0.0049)	0.3010*** (0.0138)	0.3010*** (0.0138)	0.0401*** (0.0057)	0.0398*** (0.0057)
Fn		−0.0091 (0.0072)	−0.0375*** (0.0136)	−0.0376*** (0.0136)	−0.0063*** (0.0009)	−0.0062*** (0.0009)
Fa-ag		0.0032* (0.0018)	−0.0071*** (0.0026)	−0.0071*** (0.0026)	−0.0010*** (0.0002)	−0.0010*** (0.0002)
Fpropm		−0.2410*** (0.0831)	0.1180 (0.1270)	0.1180 (0.1270)	0.0212*** (0.0048)	0.0210*** (0.0048)
Fpropp		0.0267 (0.0493)	−0.0678 (0.0932)	−0.0677 (0.0932)	−0.0072*** (0.0022)	−0.0070*** (0.0022)
Fpropw		−0.0057 (0.0615)	−0.1330 (0.0942)	−0.1340 (0.0943)	−0.0154*** (0.0034)	−0.0152*** (0.0034)
Fpropr		−0.0021 (0.0548)	0.0707 (0.0954)	0.0711 (0.0954)	0.0151*** (0.0027)	0.0150*** (0.0027)
Fay-edu		−0.0078 (0.0070)	0.0377*** (0.0102)	0.0378*** (0.0102)	0.0050*** (0.0008)	0.0049*** (0.0008)
De-log		−0.0017 (0.0028)	−0.0216*** (0.0041)	−0.0216*** (0.0041)	−0.0032*** (0.0004)	−0.0032*** (0.0004)

Standard errors in parentheses. *p < 0.1, **p < 0.05, ***p < 0.01.

From the perspective of risk assets, by comparing the results of (2), (4), (3), and (5), the coefficient of the core explanatory variable “whether to participate in urban and rural basic medical insurance” is still significant after introducing the intermediate variable of risk attitude into the original regression equation; however, its absolute value is slightly reduced, indicating that the risk attitude plays an intermediary role.

From the perspective of risk-free assets (Table 4), by comparing the results of (1), (3), (2), and (4), the coefficient of the explanatory variable “whether to participate in urban and rural basic medical insurance” is significant after the introduction of intermediary variables; but the absolute value of the variable increases slightly, indicating that the risk attitude plays a certain intermediary role. The allocation of urban and rural residents' medical insurance and NCMS has a certain negative effect on the holding probability of family risk-free assets, whereas urban residents' medical insurance has a positive impact. For the proportion of risk-free assets, the allocation of urban and rural basic medical insurance generally has a certain positive effect, which further verifies the dual role of asset substitution and the retirement effect of urban and rural basic medical insurance as a part of the social security system.

**Table 4:** Intermediary effect regression (risk-free assets).

**Explanatory variable**		**Risk-free assets**				
		**(1)**	**(2)**	**(3)**	**(4)**	**(5)**
		**Impact on rats**	**Intermediate effect**	**Direct effect**	**Intermediate effect**	**Direct effect**
			**The impact of rat on hdrfa**	**Fixed rat depends on hdrfa**	**The influence of rat on the proprfa**	**Fixed rat to look at the proprfa**
**Part 1: Medical insurance for urban and rural residents**						
	hdins	0.0217 (0.0195)		0.0170 (0.0298)		−0.0349*** (0.0033)
	Rat		−0.0106 (0.0328)	−0.0107 (0.0328)	0.0065* (0.0035)	0.0067* (0.0035)
Pri-hi		−0.0937** (0.0470)	0.0616 (0.0830)	0.0619 (0.0830)	0.0223*** (0.0057)	0.0222*** (0.0057)
Bro-sis		0.0131*** (0.0045)	−0.0133* (0.0074)	−0.0134* (0.0074)	0.0003 (0.0009)	0.0004 (0.0009)
gender		−0.1260*** (0.0230)	0.0536 (0.0354)	0.0529 (0.0353)	−0.0087** (0.0036)	−0.0075** (0.0036)
age		0.0041*** (0.0013)	−0.0048*** (0.0017)	−0.00480*** (0.0017)	0.0009*** (0.0002)	0.0008*** (0.0002)
Pri-jun edu		0.0001 (0.0572)	−0.0193 (0.0753)	−0.0222 (0.0754)	−0.0802*** (0.0098)	−0.0735*** (0.0098)
Hi-co edu		−0.1010 (0.0818)	−0.1340 (0.1160)	−0.1350 (0.1160)	−0.0614*** (0.0137)	−0.0579*** (0.0137)
Ba edu		−0.0999 (0.1060)	−0.3710** (0.1610)	−0.3700** (0.1610)	−0.0424** (0.0175)	−0.0406** (0.0175)
Y-edu		0.0097 (0.0069)	0.0258*** (0.0100)	0.0261*** (0.0100)	0.0013 (0.0011)	0.0004 (0.0011)
La		−0.0162 (0.0209)	−0.1280*** (0.0301)	−0.1300*** (0.0303)	0.0087*** (0.0031)	0.0126*** (0.0032)
M		0.1550*** (0.0417)	0.1020 (0.0708)	0.1000 (0.0708)	−0.0163* (0.0084)	−0.0158* (0.0084)
D-w		0.0781 (0.0501)	0.0781 (0.0791)	0.0769 (0.0791)	−0.0315*** (0.0098)	−0.0312*** (0.0097)
Wco		0.0070 (0.0307)	0.0061 (0.0349)	0.0049 (0.0351)	−0.0171*** (0.0040)	−0.0149*** (0.0040)
Hs		0.0314*** (0.0077)	−0.0193* (0.0117)	−0.0196* (0.0117)	−0.0083*** (0.0013)	−0.0080*** (0.0013)
Me-log		0.0015 (0.0027)	0.0094** (0.0039)	0.0095** (0.0039)	−0.0019*** (0.0004)	−0.0020*** (0.0004)
Me		−0.0001 (0.0001)	−0.0001*** (0.0001)	−0.0001*** (0.0001)	0.0001*** (0.0001)	0.0001*** (0.0001)
In-log		−0.0087** (0.0044)	0.0376*** (0.0057)	0.0378*** (0.0057)	0.0036*** (0.0009)	0.0027*** (0.0009)
As-log		−0.0137*** (0.0031)	0.1750*** (0.0050)	0.1750*** (0.0050)	−0.1390*** (0.0011)	−0.1400*** (0.0011)
Fn		0.0048 (0.0052)	−0.0249*** (0.0071)	−0.0253*** (0.0072)	−0.0005 (0.0009)	0.0007 (0.0009)
Fa-ag		0.0055*** (0.0012)	−0.0007 (0.0015)	−0.0007 (0.0015)	−0.0001 (0.0002)	−0.0001 (0.0002)
Fpropm		−0.1600*** (0.0439)	−0.1400** (0.0638)	−0.1410** (0.0638)	0.0115 (0.0074)	0.0130* (0.0074)
Fpropp		0.0137 (0.0294)	0.0673 (0.0420)	0.0674 (0.0420)	−0.0168*** (0.0052)	−0.0190*** (0.0052)
Fpropw		−0.0794** (0.0361)	0.0020 (0.0479)	−0.0008 (0.0481)	−0.0110** (0.0053)	−0.0049 (0.0054)
Fpropr		0.0331 (0.0332)	0.0733 (0.0446)	0.0741* (0.0447)	0.0135** (0.0053)	0.0117** (0.0053)
Fay-edu		−0.0082** (0.0042)	0.0209*** (0.0059)	0.0212*** (0.0059)	0.0021*** (0.0007)	0.0014** (0.0007)
De-log		−0.0024 (0.0016)	−0.0293*** (0.0025)	−0.0293*** (0.0025)	−0.0011*** (0.0003)	−0.0011*** (0.0003)
**Part 2: Medical insurance for urban residents**						
	hdins-urban	−0.0459 (0.0300)		−0.0189 (0.0421)		0.0019 (0.0041)
	Rat		0.0514 (0.0442)	0.0511 (0.0442)	−0.0072* (0.0041)	−0.0072* (0.0041)
Pri-hi		−0.0753 (0.0514)	0.00189 (0.0907)	0.00152 (0.0907)	0.0260*** (0.00614)	0.0261*** (0.00615)
Bro-sis		0.0231*** (0.0059)	−0.0125 (0.0107)	−0.0125 (0.0107)	0.0002 (0.0010)	0.0002 (0.0010)
gender		−0.159*** (0.0266)	0.0590 (0.0433)	0.0582 (0.0434)	−0.0082** (0.0040)	−0.0082** (0.0040)
age		0.0043*** (0.0016)	−0.0042 (0.0026)	−0.0042 (0.0026)	0.0013*** (0.0002)	0.0013*** (0.0002)
Pri-jun edu		0.0271 (0.0837)	0.0549 (0.1150)	0.0568 (0.1150)	−0.1100*** (0.0140)	−0.1110*** (0.0141)
Hi-co edu		−0.0673 (0.1120)	−0.0806 (0.1660)	−0.0792 (0.1660)	−0.0777*** (0.0181)	−0.0781*** (0.0181)
Ba edu		−0.0439 (0.1410)	−0.3060 (0.2230)	−0.3060 (0.2230)	−0.0401* (0.0223)	−0.0403* (0.0223)
Y-edu		0.0071 (0.0090)	0.0268* (0.0144)	0.0265* (0.0143)	−0.0010 (0.0014)	−0.0009 (0.0014)
La		−0.0169 (0.0240)	−0.0987*** (0.0373)	−0.0946** (0.0384)	0.0135*** (0.0037)	0.0132*** (0.0038)
M		0.1480*** (0.0485)	0.0807 (0.0946)	0.0813 (0.0946)	−0.0098 (0.0091)	−0.0098 (0.0091)
D–w		0.0428 (0.0597)	0.0631 (0.1060)	0.0637 (0.1060)	−0.0283*** (0.0108)	−0.0284*** (0.0108)
Wco		−0.0043 (0.0380)	0.0874* (0.0515)	0.0876* (0.0514)	−0.0305*** (0.0050)	−0.0305*** (0.0050)
Hs		0.0408*** (0.0097)	−0.0176 (0.0167)	−0.0175 (0.0167)	−0.0043*** (0.0016)	−0.0044*** (0.0016)
Me–log		0.0019 (0.0034)	0.0092* (0.0055)	0.0092* (0.0055)	−0.0022*** (0.0005)	−0.0022*** (0.0005)
Me		0.0001 (0.0001)	−0.0001 (0.0001)	−0.0001 (0.0001)	0.0001*** (0.0001)	0.0001*** (0.0001)
In-log		−0.0061 (0.0053)	0.0272*** (0.0077)	0.0270*** (0.0078)	0.0028*** (0.0010)	0.0028*** (0.0010)
As-log		−0.0170*** (0.0041)	0.1530*** (0.0071)	0.1530*** (0.0071)	−0.1560*** (0.0014)	−0.1560*** (0.0014)
Fn		0.0183** (0.0074)	−0.0226** (0.0113)	−0.0226** (0.0113)	0.0018 (0.0012)	0.0018 (0.0012)
Fa-ag		0.0060*** (0.0015)	−0.0024 (0.0021)	−0.0024 (0.0021)	0.0006*** (0.0002)	0.0006*** (0.0002)
Fpropm		−0.1420*** (0.0526)	−0.1370 (0.0847)	−0.1370 (0.0848)	0.0219** (0.0086)	0.0219** (0.0086)
Fpropp		0.0222 (0.0378)	0.1150* (0.0595)	0.1140* (0.0595)	−0.0448*** (0.0063)	−0.0447*** (0.0063)
Fpropw		−0.0744 (0.0463)	0.0015 (0.0698)	0.0003 (0.0699)	0.0066 (0.0063)	0.0067 (0.0063)
Fpropr		0.0653 (0.0425)	0.0948 (0.0633)	0.0947 (0.0634)	0.0094 (0.0064)	0.0094 (0.0064)
Fay-edu		−0.0088 (0.0054)	0.0144* (0.0086)	0.0144* (0.0086)	0.0006 (0.0008)	0.0006 (0.0008)
De-log		−0.0024 (0.0020)	−0.0233*** (0.0036)	−0.0233*** (0.0036)	−0.0012*** (0.0003)	−0.0012*** (0.0003)
**Part 3: New rural cooperative medical**						
	hdins-rural	0.0506 (0.0418)		0.1400*** (0.0522)		−0.0458*** (0.0077)
	Rat		−0.0763 (0.0473)	−0.0777 (0.0473)	0.0200*** (0.0068)	0.0202*** (0.0068)
Pri-hi		−0.1980 (0.1280)	0.2110 (0.172)	0.2120 (0.1720)	0.0361** (0.0157)	0.0362** (0.0157)
Bro-sis		−0.0048 (0.0069)	−0.0121 (0.0103)	−0.0120 (0.0103)	0.0012 (0.0015)	0.0012 (0.0016)
Gender		−0.0027 (0.0492)	0.0813 (0.0596)	0.0762 (0.0595)	−0.0048 (0.0086)	−0.00344 (0.0086)
age		0.0027 (0.0019)	−0.0046** (0.0023)	−0.0046** (0.0023)	0.0009*** (0.0003)	0.0008*** (0.0003)
Pri-jun edu		−0.0539 (0.0823)	−0.0138 (0.1030)	−0.0197 (0.1030)	−0.0690*** (0.0148)	−0.0668*** (0.0148)
Hi-co edu		−0.1300 (0.1280)	−0.0845 (0.1680)	−0.0839 (0.1680)	−0.0804*** (0.0227)	−0.0810*** (0.0227)
Ba edu		−0.3960 (0.2920)	−0.1110 (0.4080)	−0.0668 (0.4040)	−0.0924* (0.0480)	−0.1100** (0.0481)
Y-edu		0.0113 (0.0110)	0.0176 (0.0139)	0.0182 (0.0139)	0.0055*** (0.0019)	0.0053*** (0.0019)
La		−0.0670 (0.0517)	−0.2000*** (0.0708)	−0.2090*** (0.0697)	0.0248*** (0.0093)	0.0280*** (0.0093)
M		0.0507 (0.0939)	0.1190 (0.1080)	0.1030 (0.1080)	−0.0360* (0.0200)	−0.0317 (0.0200)
D-w		0.0667 (0.104)	0.0716 (0.119)	0.0571 (0.119)	−0.0401* (0.0218)	−0.0359* (0.0218)
Wco		0.0607 (0.0529)	−0.0671 (0.0494)	−0.0717 (0.0495)	−0.0034 (0.0073)	−0.0013 (0.0073)
Hs		0.0196 (0.0126)	−0.0140 (0.0163)	−0.0153 (0.0163)	−0.0119*** (0.0023)	−0.0116*** (0.0023)
Me-log		−0.0005 (0.0044)	0.0108* (0.0057)	0.0107* (0.0057)	−0.0021** (0.0009)	−0.0021** (0.0007)
Me		−0.0002** (0.0001)	−0.0001** (0.0001)	−0.0001** (0.0001)	0.0001** (0.0001)	0.0001** (0.0001)
In-log		−0.0214*** (0.0081)	0.0509*** (0.0089)	0.0506*** (0.0089)	0.0032** (0.0016)	0.0032** (0.0016)
As-log		−0.0121** (0.0049)	0.1980*** (0.0073)	0.1980*** (0.0073)	−0.1170*** (0.0022)	−0.1170*** (0.0022)
Fn		−0.0091 (0.0072)	−0.0180* (0.0096)	−0.0185* (0.0096)	−0.0019 (0.0014)	−0.0017 (0.0014)
Fa-ag		0.0032* (0.0018)	0.0022 (0.0020)	0.0023 (0.0020)	−0.0005 (0.0003)	−0.0005 (0.0003)
Fpropm		−0.2410*** (0.0831)	−0.1220 (0.0970)	−0.1180 (0.0969)	−0.0035 (0.0141)	−0.0038 (0.0141)
Fpropp		0.0267 (0.0493)	0.0308 (0.0603)	0.0243 (0.0603)	0.0097 (0.0096)	0.0111 (0.0095)
Fpropw		−0.0057 (0.0615)	0.1000 (0.0705)	0.0912 (0.0705)	0.0026 (0.0108)	0.0054 (0.0108)
Fpropr		−0.0021 (0.0548)	0.0553 (0.0632)	0.0578 (0.0632)	−0.0003 (0.0094)	−0.0014 (0.0094)
Fay-edu		−0.0078 (0.0070)	0.0209** (0.0085)	0.0211** (0.0085)	0.0003 (0.0012)	0.0002 (0.0012)
De-log		−0.00174 (0.0028)	−0.0348*** (0.0036)	−0.0348*** (0.0036)	−0.000454 (0.0005)	−0.000525 (0.0005)

Standard errors in parentheses. *p < 0.1, **p < 0.05, ***p < 0.01.

## Robustness Test

### Basic Regression Analysis

Risk assets are defined as stocks, loans, funds, derivatives, Internet financial management, financial finance, non-RMB assets, gold and other risk assets; while risk-free assets include cash, bonds, demand deposits, and time deposits. We reframe the categories of risk and risk-free assets for the robustness test; that is, for risky assets, only stocks, bonds, funds, banks, and other major items are retained, and for risk-free assets, the bonds are removed.

Column (1) in Table 5 shows that the probability of risk assets held by families with urban and rural medical insurance and urban medical insurance under the new definition increases by 0.3 and 0.5%, respectively, compared with those without corresponding insurance. Column (2) shows that under the new definition, the proportion of risk assets held by households in total assets remains basically unchanged regardless of whether the insurance is allocated.

**Table 5:** Benchmark regression (replacing definitions of financial assets).

**Explanatory variable**	**Risk assets/Risk-free assets**		**Risk assets/Risk-free assets**		**Risk assets/Risk-free assets**	
	**hdra**	**propra**	**hdra**	**propra**	**hdra**	**propra**
	**(1)**	**(2)**	**(3)**	**(4)**	**(5)**	**(6)**
**Part 1: Risk assets**						
hdins	−0.1170*** (0.0245)	−0.0160*** (0.0020)				
hdins urban			0.0203 (0.0306)	−0.0010 (0.0025)		
hdins-rural					−0.0027 (0.0599)	−0.0044* (0.0026)
Pri-hi	0.6020*** (0.0437)	0.0472*** (0.0061)	0.6510*** (0.0488)	0.0388*** (0.0071)	0.3240*** (0.1080)	0.0482*** (0.0104)
Bro-sis	0.0191*** (0.0062)	0.0007 (0.0004)	0.0156** (0.0072)	−0.0002 (0.0006)	0.0231* (0.0127)	0.0030*** (0.0007)
gender	0.0122 (0.0279)	−0.0004 (0.0023)	0.0226 (0.0305)	0.0001 (0.0028)	0.0127 (0.0748)	0.0010 (0.0028)
age	−0.0083*** (0.0015)	−0.006*** (0.0001)	−0.0057*** (0.0018)	−0.0002 (0.0002)	−0.0109*** (0.0027)	−0.00174*** (0.0003)
Pri-jun edu	−0.128 (0.0870)	−0.0249*** (0.0042)	−0.1830 (0.1180)	−0.0345*** (0.0066)	−0.0149 (0.136)	−0.0056 (0.0042)
Hi-co edu	−0.0467 (0.1150)	−0.0161** (0.0066)	−0.0919 (0.1490)	−0.0302*** (0.0098)	−0.0225 (0.196)	−0.0051 (0.0071)
Ba edu	−0.1370 (0.1450)	0.0001 (0.0095)	−0.2140 (0.1820)	−0.0175 (0.0134)	−0.1320 (0.4410)	0.0368 (0.0422)
Y-edu	0.0438*** (0.0089)	0.0040*** (0.0007)	0.0565*** (0.0108)	0.0046*** (0.0010)	0.0220 (0.0159)	0.00317*** (0.0008)
La	0.0655*** (0.0220)	0.0187*** (0.0021)	0.0524** (0.0262)	0.0113*** (0.0026)	0.0909 (0.0699)	0.0178*** (0.0044)
M	0.0845 (0.0602)	−0.0009 (0.0052)	0.1220* (0.0652)	−0.0004 (0.0065)	−0.2140 (0.1510)	−0.0351*** (0.0090)
D-w	0.0963 (0.0721)	−0.0027 (0.0057)	0.1070 (0.0793)	−0.0057 (0.0072)	−0.1130 (0.1720)	−0.0188** (0.0080)
Wco	0.0897*** (0.0316)	−0.0008 (0.0021)	0.0874** (0.0371)	−0.0042 (0.0030)	0.1700*** (0.0654)	0.0227*** (0.0044)
Hs	−0.0440*** (0.0100)	−0.0012 (0.0008)	−0.0296** (0.0118)	−0.00030 (0.0010)	−0.0746*** (0.0186)	−0.0096*** (0.0018)
Me-log	0.0053 (0.0033)	0.0001 (0.0002)	0.0049 (0.0037)	0.0001 (0.0003)	0.0092 (0.0072)	0.0017*** (0.0003)
Me	−0.0001 (0.0001)	−3.30e−08 (3.86e−08)	−0.0001 (0.0001)	−3.33e−08 (4.90e−08)	−0.0001 (0.0001)	−0.0001*** (6.87e−08)
In-log	0.1290*** (0.0097)	0.0063*** (0.0013)	0.1240*** (0.0109)	0.0029* (0.0015)	0.1360*** (0.0207)	0.0193*** (0.0033)
As-log	0.2900*** (0.0064)	0.0111*** (0.0028)	0.2820*** (0.0072)	0.0026 (0.0032)	0.3010*** (0.0137)	0.0405*** (0.00720)
Fn	−0.0578*** (0.0076)	−0.0052*** (0.0007)	−0.0635*** (0.0092)	−0.0050*** (0.0010)	−0.0364*** (0.0136)	−0.0061*** (0.0010)
Fa-ag	−0.0109*** (0.0013)	−0.0005*** (0.0001)	−0.0117*** (0.0016)	−0.0003 (0.0002)	−0.0070*** (0.0026)	−0.0010*** (0.0002)
Fpropm	0.0629 (0.0566)	0.0011 (0.0042)	0.0532 (0.0634)	−0.0043 (0.0056)	0.1180 (0.1270)	0.0206*** (0.0052)
Fpropp	0.0650 (0.0401)	0.0137*** (0.0022=)	0.1010** (0.0461)	0.0118*** (0.0034)	−0.1180 (0.0924)	−0.0135*** (0.0033)
Fpropw	−0.2100*** (0.0401)	−0.0043 (0.0032)	−0.2090*** (0.0466)	−0.0007 (0.0041)	−0.1060 (0.0943)	−0.0121*** (0.0034)
Fpropr	−0.0453 (0.0418)	−0.0048* (0.0028)	−0.0852* (0.0481)	−0.0065 (0.0040)	0.0869 (0.0956)	0.0169*** (0.0031)
Fay-edu	0.0421*** (0.0049)	0.0011** (0.0005)	0.0404*** (0.0058)	−0.0001 (0.0007)	0.0371*** (0.0102)	0.0050*** (0.0010)
De-log	−0.0103*** (0.0020)	−0.0013*** (0.0002)	−0.0062*** (0.0023)	−0.0013*** (0.0002)	−0.0219*** (0.0041)	−0.0032*** (0.0005)
**Part 2: Risk-free assets**						
hdins	0.0192 (0.0294)	−0.0310*** (0.0030)				
hdins urban			−0.0091 (0.0414)	−0.0073** (0.0037)		
hdins-rural					0.1560*** (0.0506)	−0.0120* (0.0067)
Pri-hi	0.0727 (0.0827)	0.0340*** (0.0057)	0.0077 (0.0908)	0.0301*** (0.0060)	0.2190 (0.1710)	0.0679*** (0.0156)
Bro-sis	−0.0154** (0.0073)	0.0003 (0.0007)	−0.0140 (0.0105)	−0.0003 (0.0009)	−0.0134 (0.0102)	0.0008 (0.0013)
gender	0.0489 (0.0350)	0.0035 (0.0033)	0.0481 (0.0432)	0.0043 (0.0037)	0.0837 (0.0588)	0.0176** (0.0074)
age	−0.0053*** (0.0017)	0.0005*** (0.0002)	−0.0046* (0.0025)	0.0007*** (0.0002)	−0.0049** (0.0022)	0.0003 (0.0003)
Pri-jun edu	−0.1370 (0.1140)	−0.0316*** (0.0121)	−0.0866 (0.1640)	−0.0253 (0.0159)	−0.0957 (0.1660)	−0.0581*** (0.0197)
Hi-co edu	−0.3790** (0.1590)	−0.0432*** (0.0157)	−0.3280 (0.2200)	−0.01580 (0.01990)	−0.0491 (0.4010)	−0.0988** (0.0465)
Ba edu	−0.3790** (0.1590)	−0.0432*** (0.0157)	−0.3280 (0.2200)	−0.0158 (0.0199)	−0.0491 (0.4010)	−0.0988** (0.0465)
Y-edu	0.0271*** (0.0099)	0.00339*** (0.00109)	0.0283** (0.0142)	0.0018 (0.0013)	0.0185 (0.0138)	0.0068*** (0.0017)
La	−0.1280*** (0.0296)	−0.0268*** (0.0029)	−0.0956** (0.0380)	−0.0102*** (0.0036)	−0.1910*** (0.0657)	−0.0130* (0.0076)
M	0.0996 (0.0700)	0.0178** (0.0073)	0.0995 (0.0932)	0.0153* (0.0083)	0.0838 (0.1060)	0.0086 (0.0154)
D-w	0.0789 (0.0781)	−0.0054 (0.0084)	0.0757 (0.1040)	−0.0070 (0.0096)	0.0510 (0.1180)	−0.0178 (0.0170)
Wco	0.0122 (0.0346)	−0.0129*** (0.0036)	0.0969* (0.0510)	−0.0132*** (0.0045)	−0.0638 (0.0488)	−0.0109* (0.0062)
Hs	−0.0194* (0.0116)	−0.0170*** (0.0012)	−0.0146 (0.0164)	−0.0109*** (0.0014)	−0.0170 (0.0161)	−0.0229*** (0.0020)
Me-log	0.0091** (0.0038)	−0.0007* (0.0004)	0.0086 (0.0054)	−0.0006 (0.0005)	0.0104* (0.0057)	−0.0004 (0.0007)
Me	−0.0001*** (0.0001)	0.0001*** (7.53e−08)	−0.0001 (0.0001)	0.0001*** (9.05e-08)	−0.0001** (0.0001)	−0.0001 (0.0001)
In-log	0.0379*** (0.0056)	0.0147*** (0.0008)	0.0274*** (0.0078)	0.0122*** (0.00039)	0.0506*** (0.0087)	0.0215*** (0.0014)
As-log	0.1780*** (0.0050)	−0.1360*** (0.0009)	0.1580*** (0.0069)	−0.1460*** (0.0012)	0.1990*** (0.0072)	−0.1140*** (0.0020)
Fn	−0.0248*** (0.0071)	−0.0015* (0.0008)	−0.0250** (0.0112)	−0.0002 (0.0011)	−0.0157* (0.0094)	−0.0038*** (0.0012)
Fa-ag	−0.0003 (0.0014)	−0.0005*** (0.000152)	−0.0022 (0.0021)	−0.0001 (0.0002)	0.0029 (0.0020)	−0.0005** (0.0002)
Fpropm	−0.1330** (0.0630)	−0.0016 (0.0066)	−0.1280 (0.0840)	0.0092 (0.0077)	−0.1150 (0.0953)	−0.0185 (0.0122)
Fpropp	0.0660 (0.0413)	−0.0115** (0.0046)	0.1160** (0.0589)	−0.0211*** (0.0056)	0.0159 (0.0588)	0.0138* (0.0081)
Fpropw	−0.0106 (0.0475)	−0.0196*** (0.0047)	−0.0079 (0.0692)	−0.0082 (0.0057)	0.0746 (0.0695)	0.0048 (0.0088)
Fpropr	0.0693 (0.0440)	0.0197*** (0.0047)	0.0870 (0.0625)	0.0203*** (0.0058)	0.0543 (0.0623)	0.0030 (0.0081)
Fay-edu	0.0205*** (0.0059)	0.0069*** (0.0006)	0.0127 (0.0085)	0.0051*** (0.0007)	0.0209** (0.0085)	0.0067*** (0.0010)
De-log	−0.0293*** (0.0025)	−0.0044*** (0.0002)	−0.0230*** (0.0035)	−0.0041*** (0.0003)	−0.0353*** (0.0035)	−0.0057*** (0.0005)

Standard errors in parentheses. *p < 0.1, ** p < 0.05, ***p < 0.01.

Columns (3) and (4) report that both the probability and proportion of risk-free assets held by families with urban and rural residents' medical insurance, urban residents' medical insurance, and NCMS under the new definition are basically stable compared to those without corresponding insurance types, and the main coefficients are significant at the 5% level, which is consistent with the symbol and size of the benchmark regression results.

### Intermediary Effects Tests

After adjusting the definition of financial assets, as shown in Tables 6, 7, we perform a regression analysis with risk attitude as the mediating variable to further investigate the robustness of the model.

**Table 6:** Intermediary effect regression (replacing definitions of financial assets, risk assets).

**Explanatory variable**		**Risk assets**				
		**(1)**	**(2)**	**(3)**	**(4)**	**(5)**
		**Impact on rats**	**Intermediate effect**	**Direct effect**	**Intermediate effect**	**Direct effect**
			**The impact of rat on hdra**	**Fixed rat depends on hdra**	**The influence of rat on the propra**	**Fixed rat to look at the propra**
**Part 1: Medical insurance for urban and rural residents**						
	hdins	0.0217 (0.0195)		−0.1160** (0.0246)		−0.0161*** (0.0020)
	Rat		−0.1200** (0.0249)	−0.1190** (0.0249)	−0.0122*** (0.002)	−0.0120*** (0.0024)
Pri-hi		−0.0937** (0.0470)	0.5980** (0.0438)	0.5980** (0.0437)	0.0478*** (0.0062	0.0476*** (0.0062)
Bro-sis		0.0131*** (0.00447)	0.0202*** (0.0062)	0.0204*** (0.0062)	0.0008 (0.0005	0.00083* (0.0005)
gender		−0.1260** (0.0230)	0.0019 (0.0280)	0.0064 (0.0280)	−0.0018 (0.0023	−0.0012 (0.0023)
age		0.0041** (0.0013	−0.0075*** (0.0015)	−0.0078** (0.0015	−0.0005*** (0.0001)	−0.0005*** (0.0001)
Pri-jun edu		0.0001 (0.0572)	−0.1300 (0.0877)	−0.1090 (0.0876)	−0.0264*** (0.0042)	−0.0232*** (0.0042)
Hi-co edu		−0.101 (0.0818)	−0.0384 (0.1160)	−0.0269 (0.1160)	−0.0152** (0.0066)	−0.0135** (0.0066)
Ba edu		−0.0999 (0.1060)	−0.1260 (0.1460)	−0.1180 (0.1460)	0.0022 (0.0096)	0.0030 (0.0095)
Y-edu		0.0097 (0.0069)	0.0467*** (0.0089)	0.0432*** (0.0089)	0.0044*** (0.0007)	0.0040*** (0.0007)
La		−0.0162 (0.0209)	0.0620*** (0.0221)	0.0720*** (0.0222)	0.0177*** (0.002)	0.0193*** (0.0021)
M		0.1550*** (0.0417)	0.0825 (0.0608)	0.0809 (0.0606)	−0.0007 (0.0053)	−0.00059 (0.0053)
D-w		0.0781 (0.0501)	0.0908 (0.0726)	0.0884 (0.0725)	−0.0027 (0.0058)	−0.0027 (0.0057)
Wco		0.0070 (0.0307)	0.0845*** (0.0316)	0.0900*** (0.0317)	−0.0014 (0.0021)	−0.0004 (0.0021)
Hs		0.0314*** (0.0077)	−0.0439*** (0.0101)	−0.0440*** (0.0101)	−0.0015* (0.0008)	−0.00135* (0.0008)
Me-log		0.0015 (0.0027)	0.0058* (0.0033)	0.0055* (0.0033)	0.0002 (0.0002)	0.0002 (0.0002)
Me		−0.0001 (0.0001)	−0.0001 (0.0001)	−0.0001 (0.0001)	−3.64e−08 (3.87e−08)	−3.71e−08 (3.87e−08)
In-log		−0.0087** (0.0044)	0.1310*** (0.0097)	0.1270*** (0.0097)	0.0070*** (0.0014)	0.0065*** (0.0013)
As-log		−0.0137*** (0.0031)	0.2900*** (0.0065)	0.2880*** (0.0064)	0.01170*** (0.0029)	0.0114*** (0.0030)
Fn		0.0048 (0.0052)	−0.0609*** (0.0076)	−0.0566*** (0.0076)	−0.0058*** (0.0007)	−0.0052*** (0.0007)
Fa-ag		0.0055*** (0.0012)	−0.0111*** (0.0014)	−0.0108*** (0.0014)	−0.00053*** (0.0001)	−0.0005*** (0.0001)
Fpropm		−0.1600*** (0.0439)	0.0608 (0.0571)	0.0653 (0.0570)	0.0009 (0.0042)	0.00155 (0.0042)
Fpropp		0.0137 (0.0294)	0.0865** (0.0404)	0.0725* (0.0404)	0.01530*** (0.0023)	0.0141*** (0.0023)
Fpropw		−0.0794** (0.0361)	−0.2330*** (0.0402)	−0.2180*** (0.0403)	−0.0082** (0.0034)	−0.0054* (0.0033)
Fpropr		0.0331 (0.0332)	−0.0328 (0.0420)	−0.0396 (0.0420)	−0.0044 (0.0027)	−0.0052* (0.0027)
Fay-edu		−0.0082** (0.0042)	0.0432*** (0.0050)	0.0410*** (0.0050)	0.0015*** (0.0006)	0.00115** (0.0005)
De-log		−0.0024 (0.0016)	−0.0107*** (0.0020)	−0.0108*** (0.0020)	−0.0014*** (0.0002)	−0.00137*** (0.0002)
**Part 2: Medical insurance for urban residents**						
	hdins-urban	−0.0459 (0.0300)		0.0138 (0.0308)		−0.0008 (0.0025)
	Rat		−0.1180*** (0.0281)	−0.1180*** (0.0281)	−0.0123*** (0.0031)	−0.0123*** (0.0030)
Pri-hi		−0.0753 (0.0514)	0.6510*** (0.0488)	0.6510*** (0.0488)	0.0399*** (0.0072)	0.0398*** (0.0072)
Bro-sis		0.0231*** (0.0059)	0.0165** (0.0072)	0.0166** (0.0072)	−0.0001 (0.0006)	−0.0001 (0.0006)
gender		−0.1590*** (0.0266)	0.0165 (0.0306)	0.0168 (0.0306)	−0.0006 (0.0028)	−0.0006 (0.0028)
age		0.0043*** (0.0016)	−0.0051*** (0.0018)	−0.0051*** (0.0018)	−0.0001 (0.0002)	−0.0001 (0.0002)
Pri-jun edu		0.0271 (0.0837)	−0.1550 (0.1190)	−0.1570 (0.1190)	−0.0336*** (0.0067)	−0.0336*** (0.0066)
Hi-co edu		−0.0673 (0.1120)	−0.0587 (0.1500)	−0.0596 (0.1500)	−0.0283*** (0.0100)	−0.0282*** (0.0098)
Ba edu		−0.0439 (0.1410)	−0.1750 (0.1830)	−0.1760 (0.1830)	−0.0152 (0.0134)	−0.0151 (0.0134)
Y-edu		0.0071 (0.0090)	0.0545*** (0.0108)	0.0548*** (0.0108)	0.0046*** (0.0010)	0.0046*** (0.0010)
La		−0.0169 (0.0240)	0.0597** (0.0259)	0.0573** (0.0265)	0.0114*** (0.0026)	0.0115*** (0.0026)
M		0.1480*** (0.0485)	0.1120* (0.0656)	0.1120* (0.0656)	−0.0002 (0.0065)	−0.0002 (0.0065)
D-w		0.0428 (0.0597)	0.0947 (0.0797)	0.0950 (0.0797)	−0.0058 (0.0073)	−0.0058 (0.0073)
Wco		−0.0043 (0.0380)	0.0871** (0.0372)	0.0871** (0.0372)	−0.0036 (0.0020)	−0.0037 (0.0030)
Hs		0.0408*** (0.0097)	−0.0301** (0.0119)	−0.0301** (0.0119)	−0.0004 (0.0010)	−0.0004 (0.0010)
Me-log		0.0019 (0.0034)	0.0051 (0.0037)	0.0051 (0.0038)	0.0001 (0.0003)	0.0001 (0.0003)
Me		0.000000654 (0.00000102)	−0.000000321 (0.000000680)	−0.000000318 (0.000000680)	−3.81e−08 (4.94e−08)	−3.82e−08 (4.94e−08)
In-log		−0.0060 (0.0053)	0.1210*** (0.0108)	0.1210*** (0.0108)	0.0032** (0.0015)	0.0032** (0.0015)
As-log		−0.0170*** (0.0040)	0.2800*** (0.0073)	0.2800*** (0.0073)	0.0032 (0.0032)	0.0032 (0.0032)
Fn		0.0183** (0.0074)	−0.0601*** (0.0093)	−0.0602*** (0.0093)	−0.0050*** (0.0010)	−0.0050*** (0.0010)
Fa-ag		0.0060*** (0.0015)	−0.0117*** (0.0017)	−0.0117*** (0.0017)	−0.0003 (0.0002)	−0.0003 (0.0002)
Fpropm		−0.1420*** (0.0526)	0.0560 (0.0638)	0.0558 (0.0638)	−0.0038 (0.0056)	−0.0038 (0.0056)
Fpropp		0.0222 (0.0378)	0.1110** (0.0463)	0.1120** (0.0464)	0.0128*** (0.0034)	0.0127*** (0.0034)
Fpropw		−0.0744 (0.0463)	−0.2180*** (0.0468)	−0.2170*** (0.0468)	−0.0019 (0.0042)	−0.0020 (0.0042)
Fpropr		0.0653 (0.0425)	−0.0789 (0.0483)	−0.0789 (0.0483)	−0.0073* (0.0040)	−0.0073* (0.0040)
Fay-edu		−0.0088 (0.0054)	0.0396*** (0.0059)	0.0396*** (0.0059)	0.0001 (0.0007)	0.0001 (0.0007)
De-log		−0.0024 (0.0020)	−0.0066*** (0.0023)	−0.0066*** (0.0023)	−0.0013*** (0.0002)	−0.00133*** (0.0002)
**Part 3: New rural cooperative medical**						
	Hdins-rural	0.0506 (0.0418)		0.0130 (0.0596)		−0.00241 (0.00261)
	Rat		−0.1420*** (0.0544)	−0.1370** (0.0539)	−0.0191*** (0.0040)	−0.0180*** (0.0040)
Pri-hi		−0.198 (0.128)	0.310*** (0.107)	0.343*** (0.107)	0.0456*** (0.0101)	0.0483*** (0.0106)
Bro-sis		−0.00479 (0.00693)	0.0239* (0.0128)	0.0213* (0.0127)	0.00321*** (0.000753)	0.0028*** (0.0007)
gender		−0.00267 (0.0492)	0.00457 (0.0747)	−0.0188 (0.0746)	−0.000410 (0.0028)	−0.0031 (0.0028)
age		0.0027 (0.0019)	−0.0108*** (0.0027)	−0.0111*** (0.0027)	−0.0018*** (0.0003)	−0.0018*** (0.0003)
Pri-jun edu		−0.0539 (0.0823)	−0.0193 (0.1360)	0.0012 (0.1360)	−0.0051 (0.0042)	−0.0024 (0.0042)
Hi-co edu		−0.1300 (0.1280)	−0.0291 (0.1970)	−0.0249 (0.1950)	−0.0042 (0.0071)	−0.0036 (0.0071)
Ba edu		−0.3960 (0.2920)	−0.1380 (0.4430)	−0.1370 (0.4410)	0.0402 (0.0430)	0.0396 (0.0430)
Y-edu		0.0113 (0.0110)	0.0235 (0.0159)	0.0226 (0.0158)	0.0033*** (0.0008)	0.0031*** (0.0008)
La		−0.0670 (0.0517)	0.0945 (0.0706)	0.0819 (0.0700)	0.0187*** (0.0045)	0.0173*** (0.0043)
M		0.0507 (0.0939)	−0.1890 (0.1530)	−0.1720 (0.1530)	−0.0321*** (0.0087)	−0.0292*** (0.0085)
D-w		0.0667 (0.1040)	−0.1040 (0.1740)	−0.0985 (0.1740)	−0.0176** (0.0080)	−0.0164** (0.0080)
Wco		0.0607 (0.0529)	0.1810*** (0.0654)	0.1790*** (0.0648)	0.0247*** (0.0046)	0.0241*** (0.0045)
Hs		0.0196 (0.0126)	−0.0725*** (0.0188)	−0.0767*** (0.0186)	−0.0096*** (0.0017)	−0.0100*** (0.0018)
Me-log		−0.0005 (0.0044)	0.0095 (0.0072)	0.0106 (0.0071)	0.0013*** (0.0003)	0.0014*** (0.0003)
Me		−0.0001** (0.0001)	−0.0001 (0.0001)	−0.0001 (0.0001)	−0.0001*** (6.94e−08)	−0.0001*** (6.99e−08)
In-log		−0.0214*** (0.0080)	0.1360*** (0.0208)	0.1340*** (0.0204)	0.0196*** (0.0033)	0.0189*** (0.0032)
As-log		−0.0121** (0.0049)	0.3010*** (0.0139)	0.3010*** (0.0138)	0.0412*** (0.0072)	0.0403*** (0.0072)
Fn		−0.0091 (0.0072)	−0.0401*** (0.0137)	−0.0376*** (0.0136)	−0.0067*** (0.0011)	−0.0063*** (0.0011)
Fa-ag		0.0032* (0.0018)	−0.0071*** (0.0026)	−0.0071*** (0.0026)	−0.0010*** (0.0002)	−0.0010*** (0.0002)
Fpropm		−0.2410*** (0.0831)	0.1240 (0.1280)	0.1180 (0.1270)	0.0220*** (0.0054)	0.0208*** (0.0053)
Fpropp		0.0267 (0.0493)	−0.1240 (0.0944)	−0.0677 (0.0932)	−0.0152*** (0.0034)	−0.0080*** (0.0024)
Fpropw		−0.0057 (0.0615)	−0.1010 (0.0952)	−0.134 (0.0943)	−0.0118*** (0.00338)	−0.0154*** (0.0039)
Fpropr		−0.00210 (0.0548)	0.101 (0.0966)	0.0711 (0.0954)	0.0192*** (0.00333)	0.0152*** (0.0029)
Fay-edu		−0.00782 (0.00701)	0.0366*** (0.0103)	0.0378*** (0.0102)	0.00503*** (0.000951)	0.0050*** (0.0010)
De-log		−0.0017 (0.0028)	−0.0224*** (0.0041)	−0.0216*** (0.0041)	−0.0034*** (0.0006)	−0.0032*** (0.0005)

Standard errors in parentheses. *p < 0.1, **p < 0.05, ***p < 0.01.

**Table 7:** Intermediary effect regression (replacing definitions of financial assets, risk-free assets).

**Explanatory variable**		**Risk-free assets**				
		**(1)**	**(2)**	**(3)**	**(4)**	**(5)**
		**Impact on rats**	**Intermediate effect**	**Direct effect**	**Intermediate effect**	**Direct effect**
			**The impact of rat on hdrfa**	**Fixed rat depends on hdrfa**	**The influence of rat on the proprfa**	**Fixed rat to look at the proprfa**
**Part 1: Medical insurance for urban and rural residents**						
	hdins	0.0217 (0.0195)		0.0173 (0.0298)		−0.0311^***^ (0.0030)
	Rat		−0.0085 (0.0328)	−0.0087 (0.0328)	0.0056^*^ (0.0031)	0.0058^*^ (0.0031)
Pri-hi		−0.0937^**^ (0.0470)	0.0616 (0.0830)	0.0619 (0.0830)	0.0329^***^ (0.0057)	0.0328^***^ (0.0057)
Bro-sis		0.0131^***^ (0.0045)	−0.0137^*^ (0.0074)	−0.0137^*^ (0.0074)	0.0001 (0.0007)	0.0002 (0.0007)
gender		−0.1260^***^ (0.0230)	0.0538 (0.0353)	0.0531 (0.0353)	0.0019 (0.0033)	0.0030 (0.0033)
age		0.0041^***^ (0.0013)	−0.0049^***^ (0.0017)	−0.0049^***^ (0.0017)	0.0005^***^ (0.0002)	0.0005^***^ (0.0002)
Pri-jun edu		0.0001 (0.0572)	−0.0194 (0.0752)	−0.0224 (0.0753)	−0.0354^***^ (0.0085)	−0.0295^***^ (0.0086)
Hi-co edu		−0.1010 (0.0818)	−0.1320 (0.1160)	−0.1330 (0.1160)	−0.0333^***^ (0.0122)	−0.0303^**^ (0.0122)
Ba edu		−0.0999 (0.1060)	−0.3680^**^ (0.1610)	−0.3660^**^ (0.1610)	−0.0465^***^ (0.0158)	−0.0448^***^ (0.0158)
Y-edu		0.0097 (0.0070)	0.0256^**^ (0.1000)	0.0259^***^ (0.0100)	0.0041^***^ (0.0010)	0.0033^***^ (0.0010)
La		−0.0162 (0.0209)	−0.1280^***^ (0.0301)	−0.1300^***^ (0.0303)	−0.0292^***^ (0.0029)	−0.0256^***^ (0.0029)
M		0.1550^***^ (0.0417)	0.1030 (0.0708)	0.1020 (0.0708)	0.0156^**^ (0.0074)	0.0160^**^ (0.0074)
D-w		0.0781 (0.0501)	0.0776 (0.0790)	0.0764 (0.0790)	−0.0082 (0.0085)	−0.0080 (0.0085)
Wco		0.0070 (0.0307)	0.0054 (0.0349)	0.0041 (0.0350)	−0.0157^***^ (0.0036)	−0.0137^***^ (0.0036)
Hs		0.0314^***^ (0.0077)	−0.0191 (0.0117)	−0.0194^*^ (0.0117)	−0.0171^***^ (0.0012)	−0.0168^***^ (0.0012)
Me-log		0.0015 (0.0027)	0.0093^**^ (0.0039)	0.0093^**^ (0.0039)	−0.0006^*^ (0.0004)	−0.0007^*^ (0.0004)
Me		−0.0001 (0.0001)	−0.0001^***^ (0.0001)	−0.0001^***^ (0.0001)	0.0001^***^ (7.61e−08)	0.0001^***^ (7.54e−08)
In-log		−0.0087^**^ (0.0044)	0.0374^***^ (0.0057)	0.0377^***^ (0.0058)	0.0155^***^ (0.0008)	0.0146^***^ (0.0008)
As-log		−0.0137^***^ (0.0031)	0.1750^***^ (0.0050)	0.1750^***^ (0.0050)	−0.1350^***^ (0.0010)	−0.1360^***^ (0.0009)
Fn		0.0048 (0.0052)	−0.0250^***^ (0.0071)	−0.0254^***^ (0.0072)	−0.0025^***^ (0.0008)	−0.0014^*^ (0.0008)
Fa-ag		0.0055^***^ (0.0012)	−0.0007 (0.0015)	−0.0007 (0.0015)	−0.0005^***^ (0.0002)	−0.0005^***^ (0.0002)
Fpropm		−0.1600^***^ (0.0439)	−0.1370^**^ (0.0638)	−0.1370^**^ (0.0638)	−0.0035 (0.0066)	−0.0021 (0.0066)
Fpropp		0.0137 (0.0294)	0.0676 (0.0420)	0.0677 (0.0420)	−0.0110^**^ (0.0046)	−0.0130^***^ (0.0046)
Fpropw		−0.0794^**^ (0.0361)	0.0023 (0.0478)	−0.0005 (0.0480)	−0.0247^***^ (0.0047)	−0.0193^***^ (0.0047)
Fpropr		0.0331 (0.0332)	0.0708 (0.0446)	0.0716 (0.0446)	0.0214^***^ (0.0048)	0.0197^***^ (0.0048)
Fay-edu		−0.0082^**^ (0.0042)	0.0209^***^ (0.0059)	0.0212^***^ (0.0060)	0.0074^***^ (0.0006)	0.00673^***^ (0.0006)
De-log		−0.0024 (0.0016)	−0.0293^***^ (0.0025)	−0.0293^***^ (0.0025)	−0.0044^***^ (0.0002)	−0.0044^***^ (0.0002)
**Part 2: Medical insurance for urban residents**						
	hdins-urban	−0.0459 (0.0300)		−0.0186 (0.0420)		−0.0076^**^ (0.0037)
	Rat		0.0559 (0.0442)	0.0555 (0.0442)	−0.0010 (0.00370)	−0.0011 (0.0037)
Pri-hi		−0.0753 (0.0514)	0.0020 (0.0907)	0.0016 (0.0907)	0.0295^***^ (0.0060)	0.0294^***^ (0.0060)
Bro-sis		0.0231^***^ (0.0059)	−0.0131 (0.0106)	−0.0132 (0.0106)	−0.0005 (0.0009)	−0.0005 (0.0009)
gender		−0.1590^***^ (0.0266)	0.0593 (0.0432)	0.0585 (0.0434)	0.0032 (0.0037)	0.0030 (0.0037)
age		0.0043^***^ (0.0016)	−0.0042 (0.0026)	−0.0042 (0.0026)	0.0008^***^ (0.0002)	0.00076^***^ (0.0002)
Pri-jun edu		0.0271 (0.0837)	0.0565 (0.1150)	0.0583 (0.1150)	−0.0410^***^ (0.0121)	−0.0403^***^ (0.0121)
Hi-co edu		−0.0673 (0.1120)	−0.0746 (0.1660)	−0.0732 (0.1660)	−0.0264^*^ (0.0160)	−0.0258 (0.0160)
Ba edu		−0.0439 (0.1410)	−0.2980 (0.2230)	−0.2970 (0.2230)	−0.0197 (0.0200)	−0.0192 (0.0200)
Y-edu		0.0071 (0.0090)	0.0263^*^ (0.0143)	0.0260^*^ (0.0143)	0.0019 (0.0013)	0.0018 (0.0013)
La		−0.0169 (0.0240)	−0.0992^***^ (0.0373)	−0.0952^**^ (0.0384)	−0.0105^***^ (0.0035)	−0.0092^***^ (0.0036)
M		0.1480^***^ (0.0485)	0.0830 (0.0946)	0.0836 (0.0945)	0.0155^*^ (0.0083)	0.0155^*^ (0.0083)
D-w		0.0428 (0.0597)	0.0611 (0.1060)	0.0617 (0.1060)	−0.0086 (0.0097)	−0.0086 (0.0097)
Wco		−0.0043 (0.0380)	0.0858^*^ (0.0515)	0.0860^*^ (0.0515)	−0.0132^***^ (0.0045)	−0.0132^***^ (0.0045)
Hs		0.0408^***^ (0.0097)	−0.0178 (0.0167)	−0.0176 (0.0166)	−0.0105^***^ (0.0014)	−0.0105^***^ (0.0014)
Me-log		0.00191 (0.0034)	0.0902^*^ (0.0547)	0.0090^*^ (0.0055)	−0.0006 (0.0005)	−0.0006 (0.0005)
Me		0.0001 (0.0001)	−0.0001 (0.0001)	−0.0001 (0.0001)	0.0001^***^ (9.38e−08)	0.0001^***^ (9.35e−08)
In-log		−0.0061 (0.0053)	0.0273^***^ (0.0077)	0.0271^***^ (0.0078)	0.0122^***^ (0.0009)	0.0120^***^ (0.000890)
As-log		−0.0170^***^ (0.0041)	0.1530^***^ (0.0070)	0.1530^***^ (0.0070)	−0.1460^***^ (0.0012)	−0.1460^***^ (0.00122)
Fn		0.0183^**^ (0.00738)	−0.0228^**^ (0.0113)	−0.0228^**^ (0.0113)	−0.00042 (0.0011)	−0.0004 (0.0011)
Fa-ag		0.00597^***^ (0.00151)	−0.0024 (0.0021)	−0.0024 (0.0021)	−0.0001 (0.0002)	−0.0001 (0.0002)
Fpropm		−0.142^***^ (0.0526)	−0.132 (0.0847)	−0.1320 (0.0847)	0.0080 (0.0077)	0.0081 (0.0077)
Fpropp		0.0222 (0.0378)	0.113^*^ (0.0594)	0.112^*^ (0.0594)	−0.0208^***^ (0.00557)	−0.0212^***^ (0.0056)
Fpropw		−0.0744 (0.0463)	0.00295 (0.0697)	0.00173 (0.0699)	−0.00756 (0.00570)	−0.0080 (0.0057)
Fpropr		0.0653 (0.0425)	0.0902 (0.0633)	0.0901 (0.0633)	0.0211^***^ (0.0059)	0.0210^***^ (0.0059)
Fay-edu		−0.0088 (0.0054)	0.0142^*^ (0.0086)	0.0142^*^ (0.0086)	0.0050^***^ (0.0007)	0.0049^***^ (0.0007)
De-log		−0.0024 (0.0020)	−0.0233^***^ (0.0036)	−0.0233^***^ (0.0036)	−0.0040^***^ (0.0003	−0.0040^***^ (0.0003)
**Part 3: New rural cooperative medical**						
	hdins-rural	0.0506 (0.0418)		0.1400^***^ (0.0522)		−0.0121^*^ (0.0067)
	Rat		−0.0772 (0.0473)	−0.0785^*^ (0.0473)	0.0068 (0.0057)	0.0069 (0.0057)
Pri-hi		−0.1980 (0.1280)	0.2110 (0.1720)	0.2120 (0.1720)	0.0668^***^ (0.0158)	0.0669^***^ (0.0158)
Bro-sis		−0.0048 (0.0070)	−0.0122 (0.0103)	−0.0121 (0.0103)	0.0007 (0.00128)	0.00075 (0.0013)
gender		−0.0027 (0.0492)	0.0814 (0.0596)	0.0763 (0.0595)	0.0171^**^ (0.0074)	0.0175^**^ (0.0074)
age		0.0027 (0.0019)	−0.0046^**^ (0.0023)	−0.0046^**^ (0.0023)	0.0003 (0.0003)	0.0003 (0.0003)
Pri-jun edu		−0.0539 (0.0823)	−0.0161 (0.1030)	−0.0219 (0.1030)	−0.0374^***^ (0.0127)	−0.0367^***^ (0.0127)
Hi-co edu		−0.1300 (0.1280)	−0.0873 (0.1680)	−0.0867 (0.1680)	−0.0576^***^ (0.0199)	−0.0577^***^ (0.0199)
Ba edu		−0.3960 (0.2920)	−0.1160 (0.4070)	−0.0714 (0.4040)	−0.0983^**^ (0.0468)	−0.1030^**^ (0.0469)
Y-edu		0.0113 (0.0110)	0.0176 (0.0139)	0.0183 (0.01390)	0.0070^***^ (0.0017)	0.0069^***^ (0.0017)
La		−0.0670 (0.0517)	−0.2000^***^ (0.0708)	−0.2090^***^ (0.0697)	−0.0134^*^ (0.0079)	−0.0127 (0.0079)
M		0.0507 (0.0939)	0.1180 (0.1080)	0.1030 (0.1080)	0.0044 (0.0158)	0.0057 (0.0158)
D-w		0.0667 (0.1040)	0.07210 (0.1190)	0.0576 (0.1190)	−0.0224 (0.0173)	−0.0212 (0.0173)
Wco		0.0607 (0.0529)	−0.0673 (0.0494)	−0.0719 (0.0495)	−0.0125^**^ (0.0062)	−0.0120^*^ (0.0063)
Hs		0.0196 (0.0126)	−0.0135 (0.0162)	−0.0149 (0.0163)	−0.0229^***^ (0.0020)	−0.0228^***^ (0.0020)
Me-log		−0.0005 (0.0044)	0.0107^*^ (0.0057)	0.0106^*^ (0.0057)	−0.0005 (0.0007)	−0.0005 (0.0007)
Me		−0.0001^**^ (0.0001)	−0.0001^**^ (0.0001)	−0.0001^**^ (0.0001)	−0.0001 (0.0001)	−0.0001 (0.0001)
In-log		−0.0214^***^ (0.0081)	0.0506^***^ (0.0089)	0.0503^***^ (0.0089)	0.0216^***^ (0.0014)	0.0216^***^ (0.0014)
As-log		−0.0121^**^ (0.0049)	0.1980^***^ (0.0073)	0.1980^***^ (0.0073)	−0.1140^***^ (0.0020)	−0.1140^***^ (0.0020)
Fn		−0.0091 (0.0072)	−0.0180^*^ (0.0096)	−0.0185^*^ (0.0096)	−0.0038^***^ (0.0012)	−0.0037^***^ (0.0012)
Fa-ag		0.0032^*^ (0.0018)	0.0021 (0.0020)	0.0022 (0.0020)	−0.0006^**^ (0.0003)	−0.0006^**^ (0.0003)
Fpropm		−0.2410^***^ (0.0831)	−0.1220 (0.0969)	−0.1180 (0.0969)	−0.0178 (0.0123)	−0.0180 (0.0123)
Fpropp		0.0267 (0.0493)	0.0339 (0.0603)	0.0274 (0.0602)	0.0119 (0.0082)	0.0123 (0.0082)
Fpropw		−0.0057 (0.0615)	0.0992 (0.0705)	0.0901 (0.0705)	0.0054 (0.0090)	0.0061 (0.0090)
Fpropr		−0.0021 (0.0548)	0.0542 (0.0632)	0.0567 (0.0632)	0.0024 (0.0082)	0.0021 (0.0082)
Fay-edu		−0.0078 (0.0070)	0.0211^**^ (0.0085)	0.0214^**^ (0.0085)	0.0066^***^ (0.0011)	0.0066^***^ (0.0011)
De-log		−0.0017 (0.0028)	−0.0348^***^ (0.0036)	−0.0348^***^ (0.0036)	−0.0057^***^ (0.0005)	−0.0057^***^ (0.0005)

*Standard errors in parentheses*.

* *p < 0.1,*

** *p < 0.05,*

*** *p < 0.01*.

In Table 6, Column (1) reports the influence of urban and rural basic medical insurance participation on family risk attitude under the new definition, which shows that the allocation of urban and rural residents' medical insurance and NCMS has a positive effect on risk attitude, and the allocation of urban residents' medical insurance has a negative impact on risk attitude. The symbols and absolute values of the main coefficient are in accordance with the original medium effect model.

From the perspective of risky assets, we further explore whether the results are still significant after introducing the intermediate variable of risk attitude into the regression equation under the framework of the new definition by comparing the results of Columns (2) and (4) and Columns (3) and (5) listed in Tables 3, 6. By comparing the results of the intermediary effect and direct effect, we find that although the main coefficient symbols and sizes are basically stable and the intermediary effect produced by risk attitude still exists, the absolute value of the intermediary effect and direct effect is slightly lower than that of the original intermediary effect model, which indicates that the intermediary effect of risk attitude has been weakened to a certain extent.

From the perspective of risk-free assets, by comparing the results of Columns (1) and (3) and Columns (2) and (4) listed in Tables 4, 7, we find that under the new definition, after the introduction of intermediary variables, the coefficient significance of the explanatory variable “whether to participate in urban and rural basic medical insurance” is basically stable, and the main coefficient symbols remain unchanged—all of which are significant at the 10% confidence level. However, the main coefficient of individual insurance, such as NCMS, decreases slightly, which indicates that the change in risk attitude in rural areas under the new definition has no significant impact on the proportion of household risk-free assets.

## Conclusions and Policy Recommendations

In this study, we examine the impact of the allocation of urban and rural basic medical insurance on the allocation of household liquidity risk financial assets based on the three issues of the CHFS database in 2013, 2015, and 2017. We use a Heckman two-step model to explore the impact of whether to allocate urban and rural basic medical insurance on the probability and proportion of risk assets holding empirically. Depending on the characteristics of urban-rural duality in China, we subdivide urban-rural basic medical insurance into urban-rural residents' medical insurance, urban residents' medical insurance, and NCMS for regression testing, which overcomes the interference of urban-rural heterogeneity.

First, from the perspective of risk assets, we find that the allocation of urban and rural residents' medical insurance has a negative impact on families' holding of risk assets to a certain extent, and the allocation of urban residents' medical insurance and the NCMS have a certain positive impact. All three have a negative impact on the proportion of households holding risk assets to total assets.

Second, from the perspective of risk-free assets, we find that the allocation of urban and rural residents' medical insurance, urban residents' medical insurance, and NCMS all have a certain positive impact on the probability of families' holding of risk-free assets but a certain negative impact on the proportion.

Third, overall, the allocation of basic medical insurance in urban and rural areas can promote families to make reasonable choices between risky assets and risk-free assets to a certain extent. We believe the impact mechanism may be that the allocation of urban and rural basic medical insurance increases families' risk perception, turning their risk attitude more cautious, and their investment attitude more rational. For the originally risk-seeking families, their risk asset investments are squeezed out; but for the originally risk-adverse families, their risk-free asset investments are squeezed out. Therefore, the direction of this study is consistent with the conclusion put forward by Yang and Wang (32): that China's social security system is mainly manifested as crowding-out residents' consumption and investment before it is highly improved.

Our findings show that it is not only a social and livelihood issue but also a financial field problem to effectively develop the urban and rural basic medical insurance system and provide direct empirical evidence for the crowding-out effect of residents' allocation of urban and rural basic medical insurance on the investment vitality of the domestic financial market. Further research reveals that this impact path is that urban and rural basic medical insurance reduces the proportion and possibility of households holding risk assets and risk-free assets to a certain extent by changing their risk attitude.

We thus propose two policy recommendations. First, in the process of further improving China's urban and rural basic medical insurance system, policymakers should pay attention to the impact of urban and rural basic medical insurance on the investment and consumption of families with different social and economic statuses behind the high coverage rate, and strive to make the hidden vulnerable groups enjoy more perfect protection. Second, our findings also point to the need to focus on the impact of urban and rural basic medical insurance on the investment preferences of families with different social and economic statuses behind the high coverage rate, and emphasize the positive role of urban and rural basic medical insurance systems in promoting the development of China's financial market.

In the model of this paper, we do not explore the development trends of the impact of participating urban and rural resident healthcare insurance on family ownership of financial assets over time. In future research, we can try to use measurement models such as PSM-DID models, and find the connections and progressive relations between different models, in order to obtain the inquiry results of different dimensions. For the direction of further research in the future, we believe that can be used to test whether the conclusion whose data configuration of the basic medical insurance for family financial assets choice influence is a universal in developing countries, to explore the developing countries to promote the health security system for the influence of its national household financial asset allocation and the corresponding policy recommendations.

## Data Availability Statement

Publicly available datasets were analyzed in this study. This data can be found here: https://chfs.swufe.edu.cn/datacenter/apply.

## Author Contributions

SL is responsible for data collection, data cleaning, and regression analysis. YY is responsible for introduction, institutional background, and references. The remainders of the article are responsible for both the authors equally.

## Conflict of Interest

The authors declare that the research was conducted in the absence of any commercial or financial relationships that could be construed as a potential conflict of interest.

## Publisher's Note

All claims expressed in this article are solely those of the authors and do not necessarily represent those of their affiliated organizations, or those of the publisher, the editors and the reviewers. Any product that may be evaluated in this article, or claim that may be made by its manufacturer, is not guaranteed or endorsed by the publisher.

## Footnotes

^1^Data comes from CHFS (China Household Finance Survey).

^2^Data are from *China Health Statistics Yearbook (2018)*.

^3^The medical insurance coverage of the elderly (those aged ≥ 60 years) is close to full coverage. The proportion of the elderly with at least one kind of medical insurance in the urban household registration is 92.1%, and the rural household registration is 94.0%. The coverage rate for the 45–59-year-old population is also very high, with 89% of urban residences registered and 93.8% of rural residences registered.

^4^According to the Challenge of Population Aging in China, the insurance coverage rate of poor elderly people in the urban household registration population is 6.0 percentage points lower than that of non-poor people, and the elderly living alone are 5.3 percentage points lower than those who do not. Among the rural household registration population, the coverage rate of poor elderly people is 2.6 percentage points lower than that of the non-poor, and that of elderly people living alone is 6.1 percentage points lower than that of elderly people who do not live alone.
